# The Epithelial Cell Adhesion Molecule EpCAM Is Required for Epithelial Morphogenesis and Integrity during Zebrafish Epiboly and Skin Development

**DOI:** 10.1371/journal.pgen.1000563

**Published:** 2009-07-17

**Authors:** Krasimir Slanchev, Thomas J. Carney, Marc P. Stemmler, Birgit Koschorz, Adam Amsterdam, Heinz Schwarz, Matthias Hammerschmidt

**Affiliations:** 1 Georges-Koehler-Laboratory, Max-Planck Institute of Immunobiology, Freiburg, Germany; 2 Department of Molecular Embryology, Max-Planck Institute of Immunobiology, Freiburg, Germany; 3 Koch Institute for Integrative Cancer Research, Cambridge, Massachusetts, United States of America; 4 Max-Planck Institute of Developmental Biology, Tübingen, Germany; 5 Institute for Developmental Biology, Cologne Excellence Cluster on Cellular Stress Responses in Aging-Associated Diseases, and Center for Molecular Medicine Cologne, University of Cologne, Cologne, Germany; University of Pennsylvania School of Medicine, United States of America

## Abstract

The aberrant expression of the transmembrane protein EpCAM is associated with tumor progression, affecting different cellular processes such as cell–cell adhesion, migration, proliferation, differentiation, signaling, and invasion. However, the in vivo function of EpCAM still remains elusive due to the lack of genetic loss-of-function studies. Here, we describe *epcam* (*tacstd*) null mutants in zebrafish. Maternal-zygotic mutants display compromised basal protrusive activity and epithelial morphogenesis in cells of the enveloping layer (EVL) during epiboly. In partial redundancy with E-cadherin (Ecad), EpCAM made by EVL cells is further required for cell–cell adhesion within the EVL and, possibly, for proper attachment of underlying deep cells to the inner surface of the EVL, thereby also affecting deep cell epiboly movements. During later development, EpCAM per se becomes indispensable for epithelial integrity within the periderm of the skin, secondarily leading to disrupted morphology of the underlying basal epidermis and moderate hyper-proliferation of skin cells. On the molecular level, EVL cells of *epcam* mutant embryos display reduced levels of membranous Ecad, accompanied by an enrichment of tight junction proteins and a basal extension of apical junction complexes (AJCs). Our data suggest that EpCAM acts as a partner of E-cadherin to control adhesiveness and integrity as well as plasticity and morphogenesis within simple epithelia. In addition, EpCAM is required for the interaction of the epithelia with underlying cell layers.

## Author Summary

EpCAM is a well-established marker for carcinomas of epithelial origin and a potential target for immunotherapy. In vitro analyses have implicated EpCAM in a plethora of different cellular processes, such as adhesion, motility, proliferation, differentiation, and signaling. Strikingly, depending on the context, EpCAM displayed rather opposite effects, either promoting or attenuating cell–cell adhesion versus cell migration and tissue invasion, a phenomenon described as the “double-face” of EpCAM. However, the in vivo relevance of its different effects remained largely unclear. Here, we present the first genetic analysis of EpCAM function in vivo, based on loss-of-function mutants in the zebrafish. As it is in mammals, zebrafish EpCAM is expressed in simple epithelia. Mutant embryos display defects both in epithelial morphogenesis and in epithelial integrity. Reduced epithelial morphogenesis is accompanied, and possibly caused, by an extension of apical junctional complexes and compromised basal protrusive activity. Furthermore, mutant epithelia display alterations in the relative abundance of adherence junction versus tight junction components. In addition, EpCAM tightly cooperates with E-cadherin and has a previously unrecognized trans effect on the morphogenesis and integrity of underlying cell layers. Cell differentiation and proliferation in EpCAM mutants are not, or only secondarily, affected. During later development and adulthood, EpCAM is largely dispensable, reinforcing its suitability as a target for anti-carcinoma immunotherapy with minimal side effects.

## Introduction

Like in mammalian gestation embryos, the epidermis of teleost larvae is bi-layered, consisting of an outer enveloping cell layer (EVL), which morphologically and functionally resembles the periderm of mammalian embryos [1],[2], and a basal layer of keratinocytes. The function of the mouse periderm is poorly understood, however, recent genetic evidence points to pivotal roles during skin formation and other developmental processes [3]. Furthermore, the zebrafish EVL might serve as a model for other, medically more relevant simple epithelia, such as the epithelial tubules of the developing zebrafish kidney, which during nephron morphogenesis display several crucial cellular features [4] similar to those of the EVL described here.

Zebrafish EVL cells segregate from deep cells during blastula stages and cover the embryo during further development [5], while it remains unclear whether during metamorphosis, when the zebrafish epidermis becomes stratified, they are replaced by cells derived from basal keratinocytes [6]. During gastrulation, EVL cells undergo epiboly movements to progressively spread over the yolk, tightly coordinated with the simultaneous vegetal-wards displacements of deep cells and the yolk syncytial layer (YSL) [7]–[9]. Epiboly movements start approximately 4 hours after fertilization (hpf), when only 30% of the yolk cell is covered by the blastoderm (30% epiboly), and is completed at 10 hpf, when the yolk is entirely surrounded by deep and EVL cells (100% epiboly). The molecular mechanisms underlying EVL epiboly have just started to be elucidated [10], whereas the Ca^2+^-dependent cell adhesion molecule and adherence junction (AJ) component E-cadherin (Ecad; Cdh1) has been shown to be specifically required for epiboly of deep cells [11],[12]. The major force of deep cell epiboly is their polarized intercalative displacement from inner to outer layers. According to one report, Ecad drives these directed intercalations by forming an adhesion gradient within the deep layers themselves [11], whereas according to another report, it is required for proper attachment of deep cells to the overlying EVL [12].

In addition, the EVL serves as a primary “skin”, constituting a barrier between the embryo proper and the fresh water environment. In contrast to basal keratinocytes, EVL cells are sealed to each other via apical junctional complexes (AJCs), which consist of tight junction (TJ) and, possibly, AJ sections [13]. TJ proteins like Tjp/Zo1 and Tjp/Zo3 already accumulate at the lateral/apical sides of EVL membranes during early blastula stages, while loss of Tjp/Zo3 function results in increased surface permeability and compromised osmoregulation [14]. In addition, EVL cells form desmosome-like junctions between each other and with underlying cells [12],[13].

EpCAM was first described in 1979 as a 40 KDa cell surface cancer-associated antigen [15],[16]. In addition, it was isolated in several other contexts, which resulted in a plethora of synonyms such as Tacstd1 or Trop1, recently unified under the name *Ep*ithelial *c*ell *a*dhesion *m*olecule (EpCAM) or CD326 (reviewed in [17],[18]). EpCAM is a type I single span transmembrane glycoprotein, with extracellular epidermal growth factor-like (EGF-like) and thyroglobulin (TY) motifs [17],[19],[20], and an intracellular domain containing an internalization motif and several α-actinin binding sites [19]. In mammals, EpCAM is present on the baso-lateral surface of most developing epithelia [21]–[26]. Expression is usually down-regulated as epithelial cells terminally differentiate. For example, progenitor cells of human skin epithelium express EpCAM, whereas differentiated keratinocytes do not [22]. However, EpCAM levels often rise again during regeneration or neoplastic transformations [18], [23], [24], [27]–[29]. Strikingly, EpCAM is only found in epithelial-derived cancers, i.e., carcinomas, but not in others, such as sarcomas, melanomas, or lymphomas [30].

Despite exhaustive in vitro studies, the exact roles of EpCAM during tumor progression and the molecular and cellular mechanisms of its functions are not fully understood, with several controversial findings, implicating EpCAM with adhesion, migration, metastasis, proliferation, differentiation, signaling and metabolism (reviewed in [18],[31]). Whereas according to some data, EpCAM acts as a homophilic cell-cell adhesion molecule with positive effects on cell adhesiveness and negative effects on cell motility and metastasis [26], [32]–[34], other data are in line with anti-adhesive and migration-promoting functions of EpCAM. Thus, in the presence of classical cadherins, EpCAM can reduce cell-cell adhesion, possibly by interfering with the interaction between cadherins and the cytoskeleton [20],[35]. In addition, EpCAM physically interacts with the metastasis-promoting cell surface receptor CD44v4-V7 [36] and the tight junction component Claudin7 [37], possibly blocking Claudin function during invasion and metastasis of several carcinomas [38],[39]. Furthermore, EpCAM enhances proliferation rates of carcinoma cells [34],[40], presumably mediated via direct nuclear signaling of its proteolytically cleaved intracellular domain EpICD [41], and via c-Myc and the cell cycle regulators cyclin A and E [42]–[44].

Most of these functional data were obtained in cell or tissue culture systems and via EpCAM overexpression. In contrast, in vivo and loss of function studies are scarce. No EpCAM mouse mutants have been reported so far. In zebrafish, EpCAM (Tacstd) mutants were generated via random retroviral insertions, characterized by delayed otolith formation in the developing inner ears [45]. In this study we generate maternal-zygotic zebrafish EpCAM mutants and chimeric embryos, revealing essential roles of EpCAM in the EVL for proper epithelial morphogenesis integrity during epiboly and skin development. Some of these roles are fulfilled in partial or complete redundancy with E-cadherin, whereas for others, EpCAM is absolutely indispensable. The molecular mechanisms underlying these in vivo functions are not completely clear. However, mutant cells display basal extensions of TJs and increased membrane levels of TJ components, coupled with reduced Ecad membrane localization and reduced protrusive basal activity. After skin formation, mutants also display hyper-proliferation of EVL and the underlying epidermal cells, which, however, seems to be a secondary consequence of the epithelial defects. We conclude that EpCAM acts as cell-cell adhesion molecule and a partner of E-cadherin, promoting both epithelial integrity and epithelial morphogenesis.

## Results

### *epcam* zebrafish mutants were identified in screen for abnormal skin development

**Figure 1:**
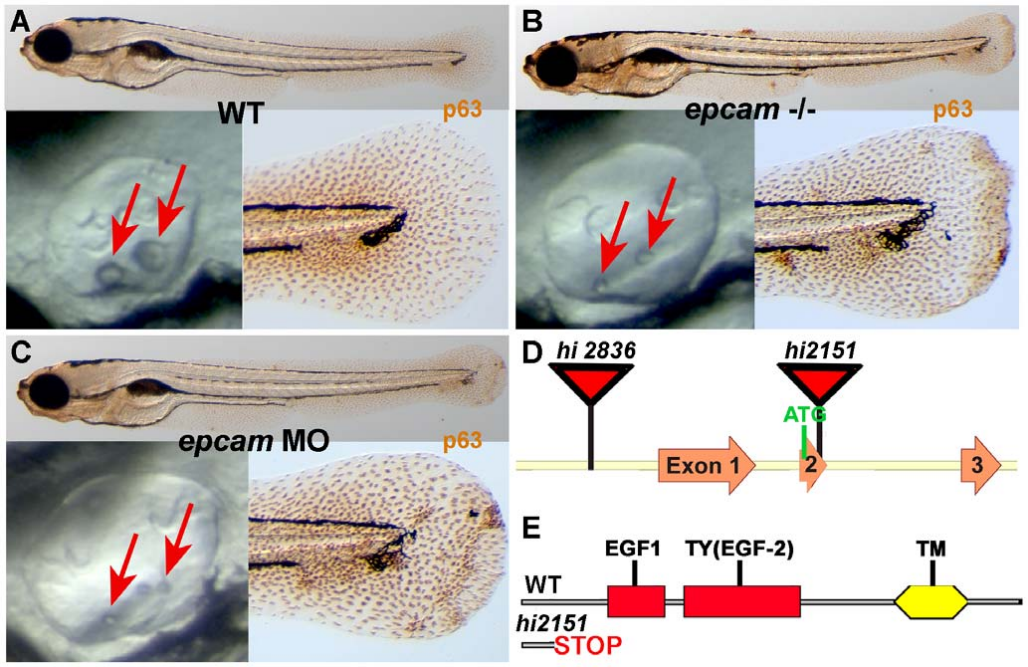
*epcam* mutants and morphants display smaller otoliths and keratinocyte aggregation. Panels (A–C) show wild-type siblings (WT, A), zygotic (M+/−,Z−/−) *epcam* mutants (*epcam* −/−) and *epcam* morphants (*epcam* MO); upper panels show larvae at 120 hpf after anti-p63 immunostainings of basal keratinocytes, lower right panels show magnification of tail tip regions, and lower left panels show lateral views on otic vesicles of live embryos at 48 hpf. Red arrows point to otoliths, which are smaller in mutants, but which recover later (see Figure S1). (D,E) Schematic illustration of the two *epcam* alleles generated by retroviral insertional mutagenesis [45]. In *hi2836* the retroviral cassette is inserted upstream of exon1 (D), disrupting *epcam* transcription. In *hi2151* the insertion is 42 base pairs downstream of the translational initiation site, introducing termination codons in all reading frames and leading to a premature termination of the protein that removes all described functional domains of EpCAM (EGF, epidermal growth factor-like domain; TY, thyroglobulin domain; TM, transmembrane domain) (D,E).

To identify genes with essential functions during zebrafish skin development, we performed an antibody-based screen on a previously described bank of retroviral insertional mutants [45], staining larvae at 120 hours post fertilization (hpf) for the basal keratinocyte-specific transcription factor ΔNp63, which is required for epidermal development in both fish and mammals [46]–[51]. The bank contained two mutant alleles of *epcam* (ZFIN: *tacstd*; GenBank accession number NM212175), *hi2151* and *hi2836*, which were described to have delayed otolith development (Figure 1A and 1B) [45]. In the case of *hi2836*, the retroviral cassette is inserted upstream from exon 1 of the EpCAM gene, whereas the insertion in *hi2151* is within exon 2, causing a frame shift and premature termination of the protein that removes all annotated functional domains (Figure 1D and 1E). In our assay, both alleles displayed aggregates of basal keratinocytes of undistinguishable strengths (Figure 1A and 1B; and data not shown). Similar aggregates, as well as the characteristic delay in otolith development, were observed in embryos injected with an antisense morpholino oligonucleotide (MO) targeting the translational start site of *epcam* mRNA (Figure 1C).

*epcam* mutants kept under standard conditions (28°C) usually died between day 5 and 7 of development, whereas they were sub-viable when kept under semi-sterile conditions and at lower temperature (25°C). However, keratinocyte aggregates of survivors remained visible throughout the first three weeks of development, and fish grew more slowly (Figure S1B, S1C). Adult homozygous mutants were fertile and appeared morphologically normal, even in histological studies (data not shown).

### Maternal *epcam* transcripts are ubiquitously distributed, whereas zygotic expression is restricted to epithelial structures, including the skin

**Figure 2:**
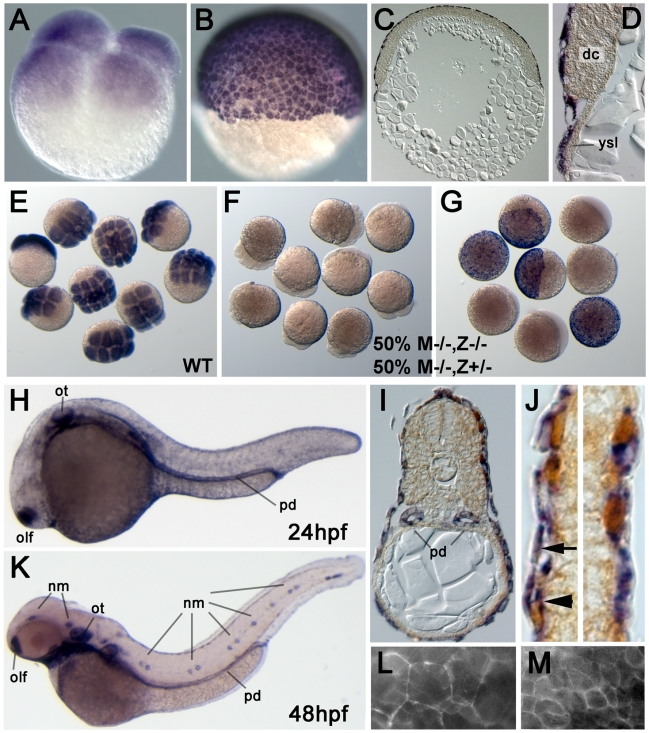
Zebrafish *epcam* is expressed in multiple epithelia, including the skin. (A–J) In situ hybridizations with anti-sense *epcam* probe. (A) Maternally provided *epcam* RNA is uniformly distributed in all blastomeres of 4-cell stage embryo (1 hpf). (B–D) At the 50% epiboly stage (early gastrula; 5.5 hpf), *epcam* expression is restricted to the outer EVL layer. (C,D) show 8 µm thick paraffin sections; in (D) *epcam*-negative deep cells (dc) and yolk syncitial layer (ysl) are indicated. (E–G) Mutant *epcam* mRNA is unstable, and zygotic *epcam* transcription is initiated before gastrulation. (E) Wild-type (WT) embryos at 8–16 cell stage; (F,G) embryos from cross of homozygous mutant female (M−/−) and heterozygous male, 50% of which are zygotic heterozygotes (Z+/−), and 50% zygotic homozygous mutants (Z−/−). At the 8–16 cell stage (F), all embryos lack (maternally supplied) *epcam* transcripts. At the 50% epiboly stage (G), Z−/− embryos still lack *epcam* mRNA, whereas zygotically derived transcripts are detectable in Z+/− embryos, indicating that zygotic *epcam* transcription starts shortly after midblastula transition [88]. (H–K) At 24 hpf (H–J) and 48 hpf (K), *epcam* is expressed in the olfactory placodes (olf), otic vesicles (ot), head and lateral line neuromasts (nm), pronephric ducts (pd) and the skin; (I,J) transverse sections through trunk of 24 hpf embryo labeled for *epcam* RNA (in blue) and p63 protein (in brown). *epcam* is expressed both in the (p63-negative) outer EVL (arrow in J), and in the underlying layer of p63-positive basal keratinocytes (arrowhead in J). (L, M) Epifluorescent images of live embryo at 9 hpf. Upon injection of mRNA encoding EpCAM-GFP, the fusion protein localizes to the cell membrane of both EVL cells (L) and the underlying deep cells (M). Note the size differences between the two cell types.

*epcam* was previously shown to be expressed in migrating neuromast primordia, otic vesicles and olfactory placodes [52]–[54]. Here, we have extended these analyses with special focus on the skin. Maternally provided *epcam* mRNA was uniformly distributed in all cells of cleavage and early blastula stage embryos (Figure 2A). After the onset of epiboly, the first of the morphogenetic movements of gastrulation, during which the yolk becomes progressively overgrown by the blastoderm [7], *epcam* mRNA was restricted to the enveloping layer (EVL), whereas deep cells had become *epcam*-negative (Figure 2B–2D). In offspring of homozygous *hi2151* or *hi2836* mothers and heterozygous fathers, all embryos lacked *epcam* transcripts at cleavage stages (compare Figure 2F with Figure 2E), whereas at early gastrulation, only 50% (most likely M−/−,Z−/−; see below) remained negative, while the other half (M−/−,Z+/−) had gained normal EVL expression (Figure 2G). This indicates that zygotic *epcam* expression starts at blastula stages. In addition, it suggests that the *hi2836* and *hi2151* mutations cause a transcriptional blockade or mRNA instability, respectively, and that both alleles are null mutations.

At 24 and 48 hours post fertilization (hpf), in addition to the previously described epithelial structures (see above; Figure 2H and 2K), we noticed persistent *epcam* expression in the EVL and expression in the basal epidermis (Figure 2I and 2J), a derivative of the ventral ectoderm which during gastrula and early segmentation stages had been *epcam*-negative (Figure 2C). This expression persisted throughout the investigated larval stages (until 120 hpf). Also, according to RT-PCR analyses, *epcam* RNA was present in the skin of adult zebrafish (data not shown). To investigate the subcellular distribution of EpCAM protein, we tried immunostainings with different antibodies against human or mouse EpCAM (see Materials and Methods), however, none of them gave specific signals (data not shown). Therefore, we injected early zebrafish embryos with mRNA encoding full-length zebrafish EpCAM fused to Green fluorescent protein (GFP). The fusion protein was detected at the cell membrane of both EVL and deep cells (Figure 2L and 2M).

### Maternal-zygotic EpCAM is primarily required in the EVL

**Figure 3:**
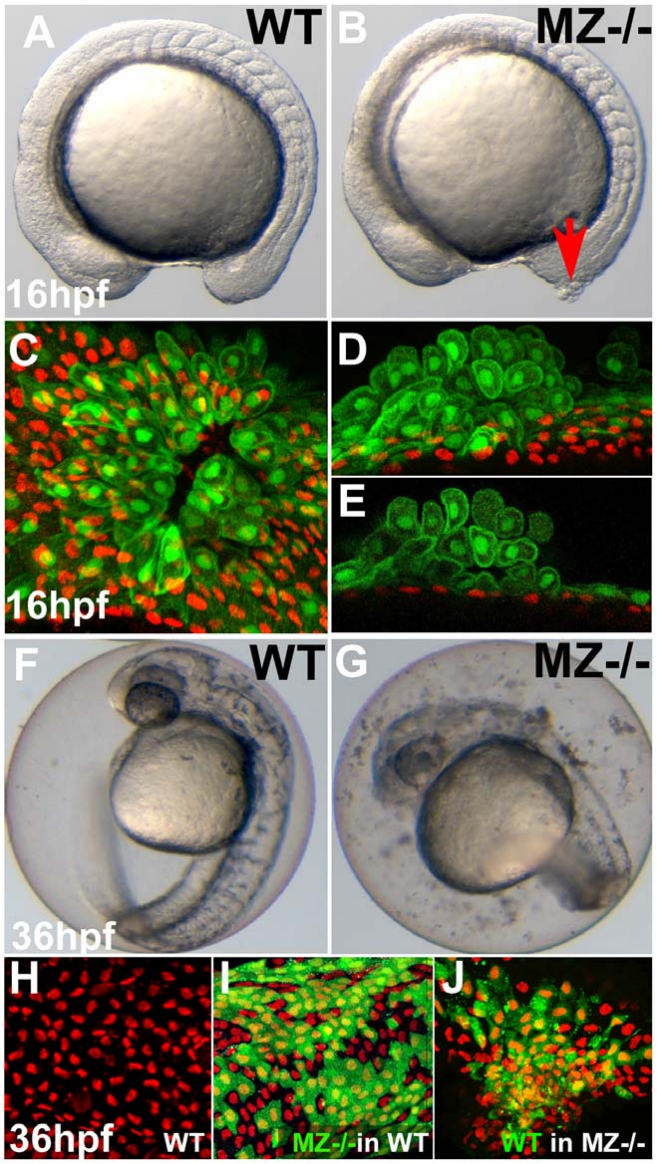
First cell aggregates of maternal/zygotic *epcam* mutants are formed during mid segmentation stages, starting in the EVL, whereas basal cell aggregates form secondarily. (A,B) Maternal/zygotic (MZ) *epcam* mutant at 16 hpf (12-somite stage) (B) with skin aggregate on tailbud (red arrow). (C–E) Confocal images of skin aggregate at 16 hpf; (C) merged stack of sagittal sections; (D) merged stack and (E) single layer of transverse sections; EVL cells labeled in green (anti-GFP immunostaining of tg(*cytokeratin8:GFP*) product), nuclei of basal keratinocytes in red (anti-p63 immunostaining). The EVL is ruptured (C), EVL cells have rounded up and have piled up on each other (D,E). In contrast, the underlying basal layer is normally organized, with regularly spaced p63-positive nuclei (D,E). (F,G) Live images of un-hatched embryos at 36 hpf. Sloughed skin cells are present within the chorion of mutant embryo (G). (H–J) Aggregates of basal keratinocytes are formed as a consequence of the loss of EpCAM function in the EVL; stacks of confocal images; transplanted basal cells are stained for GFP in green, nuclei of basal cells for p63 in red. (H) Non-chimeric wild-type control. (I) Chimeric embryo with cluster of MZ*epcam* mutant basal cells (in green) in wild-type environment/underneath wild-type EVL, with normal spatial organization of mutant basal cells. (J) Chimeric embryo with cluster of wild-type basal cells (in green) underneath MZ*epcam* mutant EVL, with aggregations of wild-type basal cells.

Early zebrafish development is regulated by a combination of maternal factors (deposited in the egg during oogenesis) and zygotic factors generated by the embryo itself. Crossing homozygous or heterozygous *epcam* mutant females with heterozygous males, we could generate mutants of three different genotypes: M+/−,Z−/−, which still have the maternally supplied *epcam* gene products, but lack the embryonic contribution (zygotic effect), M−/−,Z+/−, which lack the maternal, but contain embryonic gene products (maternal effect), and M−/−,Z−/−, which lack both the maternal and the embryonic supply (maternal-zygotic effect; MZ−/−). By all means M−/−,Z+/− mutants had wild-type appearance, indicating that zygotic *epcam* gene products are sufficient for normal development. Nevertheless, defects of MZ−/− embryos were more severe and developed earlier than in zygotic mutants, indicating that maternally supplied gene products can partly take over the role of zygotic *epcam*: whereas skin aggregates in zygotic mutants only developed during the second day of development (data not shown), in MZ−/− mutants, they were already apparent at mid-segmentation (16 hpf; Figure 3A and 3B), while later, skin aggregates were larger (Figure S1A), and high numbers of shed skin cells were found floating in the chorion (starting at approximately 16 hpf; see Figure 3G for 36 hpf). At 16 hpf, aggregates often occurred in the tailbud region, where the tail grows out. Here, the skin is most likely exposed to highest mechanical pressure and/or undergoes most dramatic epithelial morphogenesis (Figure 3B). To investigate cell aggregates in greater detail, we took advantage of a transgenic zebrafish line in which EVL cells are labeled by GFP [55]. Our analysis showed that the early skin aggregates in MZ−/− embryos primarily consisted of EVL cells, which had acquired a roundish shape and had piled up on each other. In contrast, p63-positive basal cells underneath the foci seemed unaltered (Figure 3C–3E), and only formed aggregates much later (see Figure S1A for 48 hpf). At these later stages, *epcam* is expressed both in EVL and basal cells (see above; Figure 2J). To distinguish whether the late aggregation of basal cells is caused by a loss of EpCAM in basal cells themselves, or by its loss in the overlying EVL cells, we generated chimeric embryos, transplanting basal MZ−/− cells into wild-type hosts and vice versa [56]. Strikingly, even largest clones of mutant basal cells were organized normally when present in a wild-type environment (compare Figure 3I with Figure 3H), whereas clones of wild-type basal cells in mutant hosts formed aggregates indistinguishable from those in non-chimeric mutants (Figure 3J). In sum, this indicates that EpCAM from EVL cells is required for proper epithelial organization both within the enveloping layer per se and in the underlying layer of basal keratinocytes.

### *epcam* mutants exhibit secondary increase in EVL and epidermal proliferation

**Figure 4:**
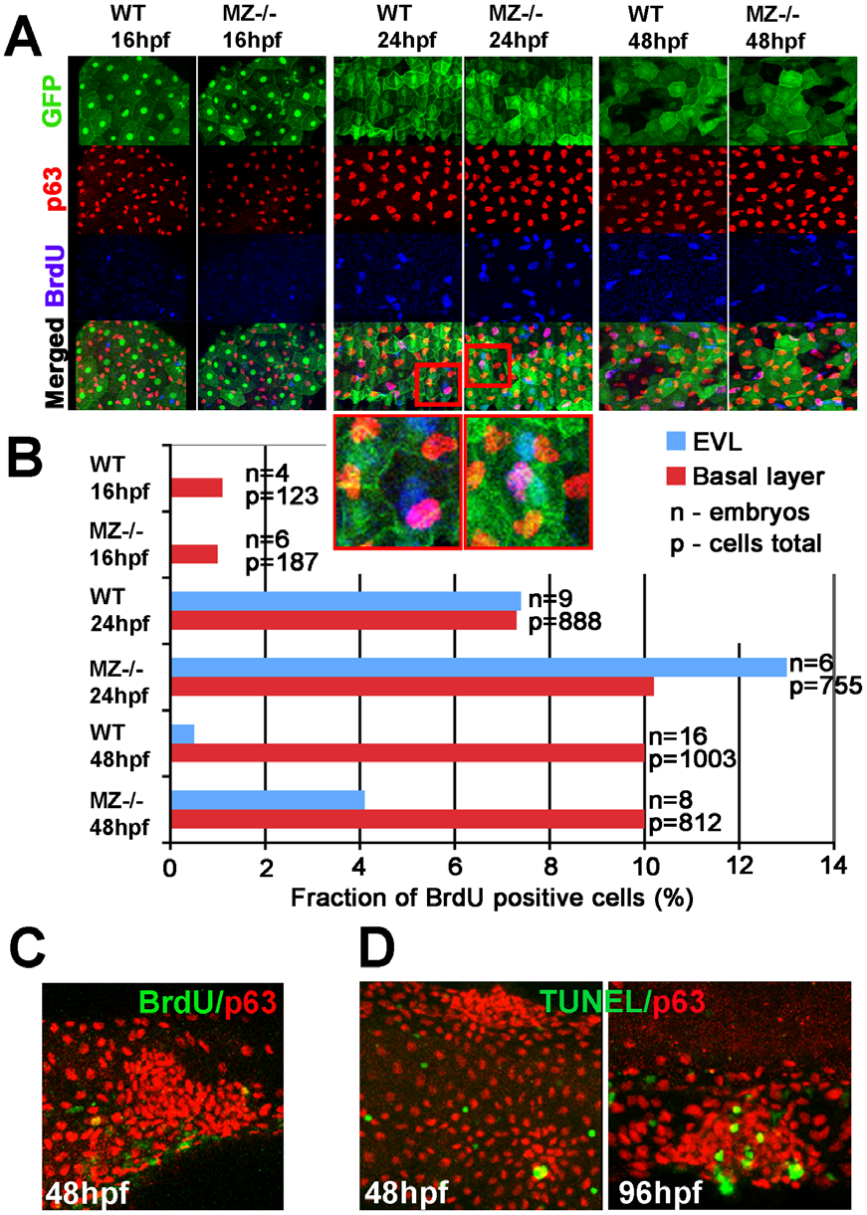
MZ*epcam* mutants display secondary increase in proliferation and apoptosis of EVL cells and basal keratinocytes. (A) Confocal images of maternal/zygotic *epcam* (MZ−/−) mutant and wild type (WT) embryos at indicated stages. EVL cells and their nuclei are in green (anti-GFP immunostaining of tg(*cytokeratin8:GFP*) product; row 1), nuclei of basal keratinocytes in red (anti-p63 immunostaining; row 2), and BrdU-positive nuclei in blue (row 3). Row 4 shows merged images; row 5 magnified views of regions boxed in row 4 in red. They show examples of BrdU-positive EVL (in light blue; green+blue) and basal cell nuclei (in purple; red+blue). (B) Percentages of BrdU-positive EVL (in blue) and basal layer (in red) nuclei in WT and MZ−/− embryos at the indicated stages of development. (C) Confocal image of aggregate of p63-positive basal cells (in red) at 48 hpf; the aggregate is devoid of BrdU incorporation (in green), whereas BrdU-positive nuclei are present outside the aggregate, indicating that the assay worked. (D) Analysis of apoptosis levels within epidermal aggregates of MZ−/− embryos. At 48 hpf, aggregates contain very few or no TUNEL-positive cells (green), while more apoptotic cells are found in aggregates at 96 hpf.

Elevated EpCAM levels in transformed tissues are thought to promote cell proliferation, contributing to carcinoma progression [34],[40]. To determine proliferation rates in zebrafish *epcam* mutants, we carried out Bromodeoxyuridine (BrdU) incorporation studies in combination with EVL or epidermal-specific markers [56]. During somitogenesis (16 hpf), when EVL aggregation and cell shedding is already apparent, we could not detect any difference in the number of BrdU labeled cells between mutants and their WT siblings (Figure 4A and 4B). At 24 hpf, proliferation rates in both the EVL and the basal layer of *epcam* mutants were slightly elevated, while at 48 hpf, this difference only persisted in the EVL, but not in the basal layer (Figure 4A and 4B). Strikingly, aggregates of mutant EVL or basal cells displayed BrdU incorporation rates as in non-affected regions of the skin (Figure 4C; and data not shown). Together, this suggests that cell aggregates in *epcam* mutants are not due to hyper-proliferation and that, in turn, hyper-proliferation might be a secondary consequence of compromised epithelial integrity.

### EpCAM mutants display higher infection susceptibility and enhanced skin inflammation

At 24 hpf and later, we also observed increased numbers of leukocytes in the skin of MZ*epcam* mutants (Video S1, Video S2; and data not shown), similar to the recently described defects in zebrafish mutants lacking the Hepatocyte growth factor activator inhibitor Hai1 [56],[57]. However, genetic ablation of the myeloid lineage in MZ*epcam* mutants with *pu.1* antisense MOs [58] did not ameliorate the skin defects, ruling out that they are secondary consequences of enhanced skin inflammation (data not shown). In *hai1* mutants, skin invasion by innate immune cells might be triggered by the apoptosis of skin cells [56]. However, according to TUNEL stainings, even the skin aggregates of EpCAM mutants only displayed moderately increased numbers of apoptotic cells after 48 hpf (Figure 4D; data not shown), making such a mechanism rather unlikely. To test whether leukocytes are activated as a result of compromised skin barrier and enhanced infection, we compared wild-type and mutant embryos kept under semi-sterile conditions or after incubation in water contaminated with bacteria. Under semi-sterile conditions, wild-type and mutant embryos displayed identical patterns of leukocyte distribution, revealed via *leukocyte-specific-plastin (lcp1)* [59],[60] in situ hybridization at 48 hpf (Figure S2A, S2B). However, after bacterial challenge for 2 hours, the skin of *epcam* mutants contained much more inate immune cells (Figure S2D) than in challenged wild-type controls (Figure S2C) or un-challenged mutant siblings (Figure S2B). In sum, these data suggest that loss of EpCAM primarily affects epithelial properties of the enveloping cell layer, whereas dysmorphology of the basal layer, hyper-proliferation, apoptosis, infection and inflammation are later or secondary consequences.

### Loss of *epcam* causes compromised protrusive activity and morphogenesis in EVL cells during epiboly

**Figure 5:**
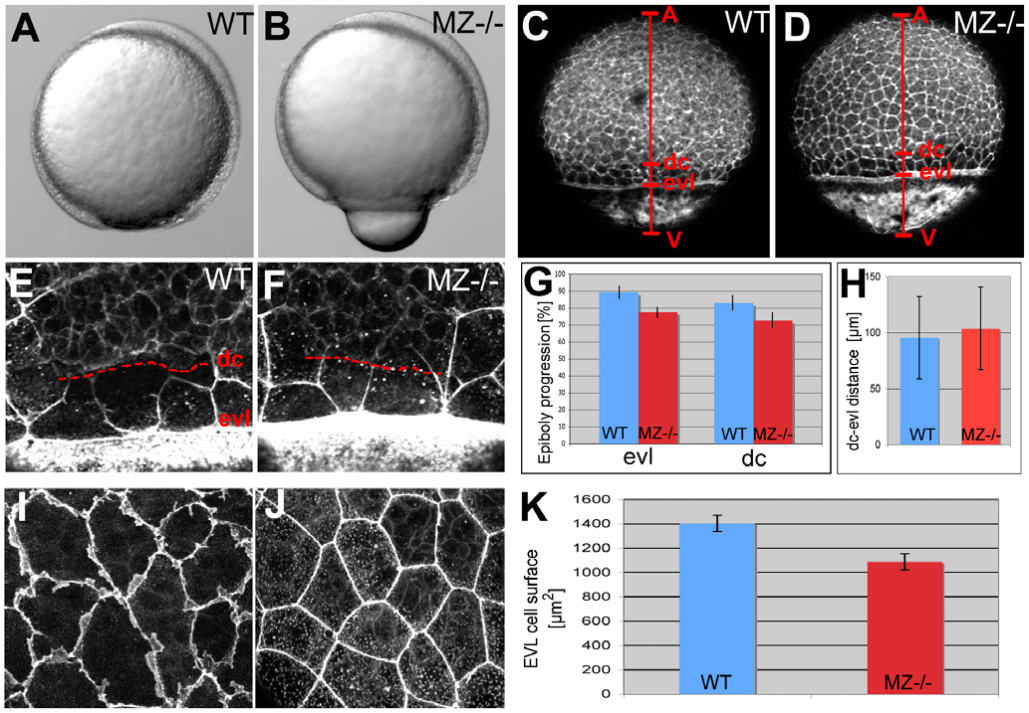
EpCAM is required for EVL and deep cell layer epiboly. (A,B) Live animals at the 90% epiboly stage, dorsal to the right; (C–F,I,J) phalloidin stainings of the actin cytoskeleton at 80% epiboly. (A,B) One of the morphogenetic processes during epiboly is the constriction of marginal EVL cells [10]. In mutants, this constriction does occur, however, at aberrant, slightly more animal positions, leading to a protrusion of the yolk plaque (B). (C,D) Overview over entire embryos; red bars indicate the animal (A) - vegetal (V) axis, and the margins of the deep cell layers (dc) and the enveloping layer (evl). The mutant (D) displays slightly retarded epiboly of the EVL and the deep cells (see larger distances between the evl margin and the vegetal pole (evl-V) and between the dc margin and the vegetal pole (d-V) compared to wild-type control in C). (G) Graphic demonstration of average progression of evl and dc epiboly in MZ−/− mutants and wild-type controls (WT). Percentage values were calculated from images like in (C,D) as (distance A-evl)/(distance A-V) or (distance A-dc)/distance (A-V). Standard deviations are indicated. For exact numbers see Text S1. (E,F) Magnified views on the evl and dc margins; dc margins are indicated by dashed red lines. (H) Graphic demonstration of average evl-dc distances in MZ−/− mutants and WT controls, as shown in (E,F). n = 5; standard errors are indicated. (I,J) Magnified views on EVL in regions slightly animal of the deep cell border (merged Z-stacks of confocal images), revealing ruffle-like protrusions in the wild type (I) that are missing in the mutant (J). The presence of intracellular, vesicular-like phalloidin staining varied from embryo to embryo, and even from cell to cell, but seemed unrelated to the *epcam* genotype. (K) Graphic demonstration of average horizontal sizes of EVL cells of WT and MZ−/− embryos at 80% epiboly, as shown in (E,F). n = 30; standard errors are indicated.

**Figure 6:**
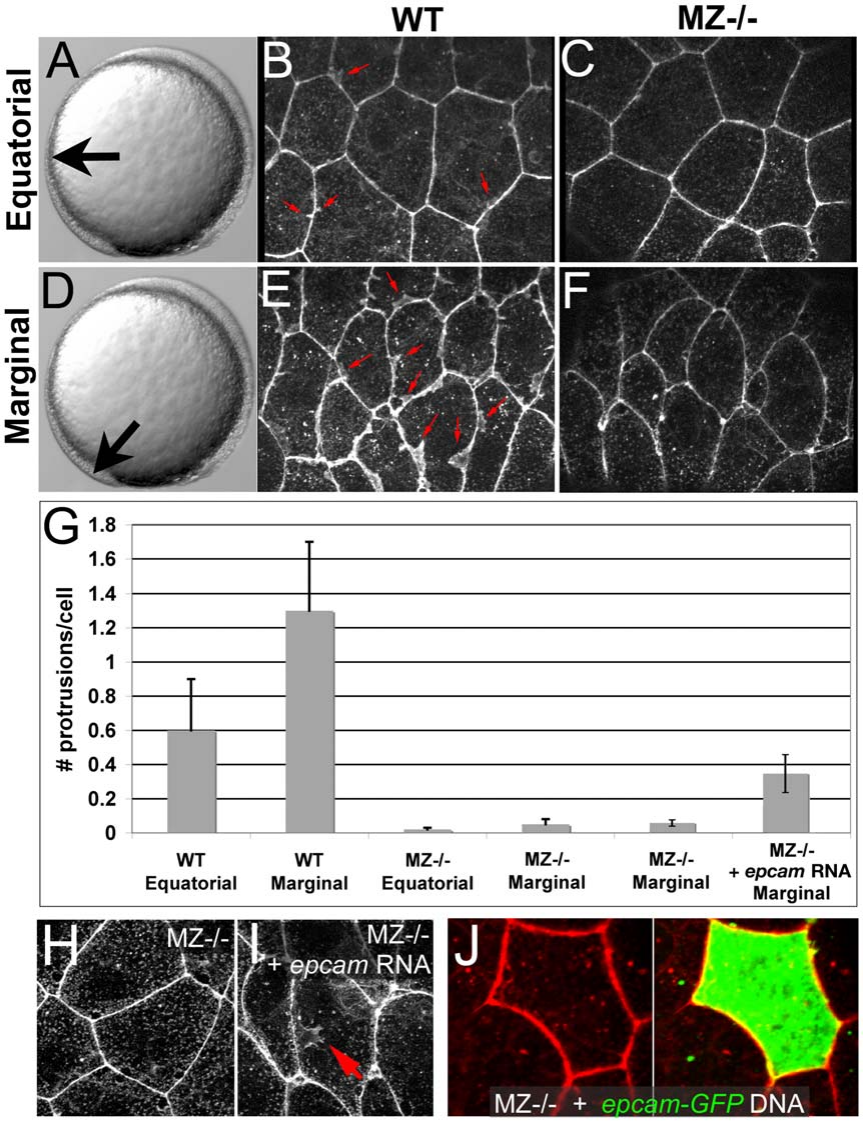
Ruffle formation in marginal EVL cells is partly restored upon uniform re-introduction of *epam* via RNA injection, whereas *epcam* overexpression in single EVL cells is insufficient. (A–F) Phalloidin stainings of wild type (WT) and maternal zygotic EpCAM mutant (MZ−/−) EVL cells at two different positions, equatorial (A–C) and marginal (D–F); merged Z-stacks of confocal images. In WT embryos, marginal EVL cells are much more protrusive than equatorial EVL cells, while in MZ−/− embryos, EVL cells show practically no protrusive activity at both locations. First four columns of panel (G) show quantification of protrusive activity. (H,I) Phalloidin stainings of marginal EVL cells from un-injected maternal/zygotic EpCAM mutant (MZ−/−) (H) and MZ−/− mutant injected with zebrafish *epcam* mRNA (I), 80% epiboly stage. There was a partial restoration of the protrusive activity of the EVL cells (red arrow in H), as quantified in (G), columns 5 and 6. Injection of higher amounts of *epcam* mRNA caused progressive death of injected embryos during pre- or early gastrula stages (data not shown), most likely due to ectopic effects of applied EpCAM in the deep cells, which precluded analyses of the EVL phenotype. (J) Injection of plasmid DNA encoding EpCAM-GFP fusion protein under the control of CMV promoter in MZ−/− embryos led to high recombinant protein levels in single cells. In most cases, this also caused death of expressing cells (data not shown). The few surviving EVL cells failed to restore ruffles (n = 0/13), suggesting that ruffle formation also requires EpCAM function in adjacent EVL cells, to which the protrusions usually attach (compare with Figure 7). Unfortunately, in over 100 investigated embryos, we failed to obtain clones with adjacent EpCAM-GFP-positive EVL cells, as would have been necessary to directly test this notion.

The EVL-specific defects of MZ*epcam* mutants during segmentations stages prompted us to carry out more thorough analyses of this cell lineage during earlier development. During epiboly, cells throughout the EVL flatten out and increase their apical and basal surfaces [8],[9], while marginal EVL cells constrict, involving actin and myosin 2 that is localized in the yolk cytoplasm along the margin of the EVL [10]. In MZ*epcam* mutant embryos, this constriction occurred at positions slightly closer to the animal pole than in wild-type embryos, resulting in an extrusion of the vegetal-most part of the yolk at mid gastrula stages (Figure 5A and 5B) and, sometimes, in embryonic death. This suggests that loss of EpCAM might specifically affect the vegetal-wards spreading of the EVL, but not the actin-myosin-dependent constriction of marginal EVL cells. Compromised EVL epiboly as a result of reduced constrictions at the EVL margin has recently been described for embryos after knock-down of *msn1*, the zebrafish ortholog of the *Drosophila* Ste20-like kinase Misshapen that is required for actin/myosin 2 recruitment, or after treatment with the specific myosin 2 inhibitor blebbistatin [10]. In contrast, phalloidin stainings of the actin cytoskeleton revealed normal constrictions of marginal EVL cells in MZ*epcam* mutants (Figure S3). However, MZ*epcam* mutants did display a significant reduction in yolk coverage by the EVL at late gastrula stages (77±4.9% (n = 9) versus 89±4.7% (n = 10) in wild-type embryos of the same age; Figure 5C, 5D, and 5G), and a corresponding reduction in the average surface of individual EVL cells (Figure 5I, 5J, and 5K). Epiboly of deep cells was similarly delayed in MZ*epcam* mutants (72±4.8% (n = 9) versus 83±3.1% (n = 10) in wild type embryos; Figure 5C, 5D, and 5G), while the distance between the marginal borders of the EVL and the deep cell layer was as in wild-type embryos (Figure 5E, 5F, and 5H). Furthermore, and most strikingly, whereas the lateral sides of marginal and, to a lower extent, equatorial wild-type EVL cells appeared ruffled, indicating the presence of (basal; see below) cellular protrusions, MZ*epcam* mutant cells lacked these ruffles (Figures 5I and 5J and Figure 6A–6G). Ruffles could be at least partly restored by injection of synthetic *epcam* mRNA into mutant embryos (Figure 6G–6I). However, no rescue was obtained upon *epcam* re-introduction into single EVL cells (Figure 6J; n = 0/13), suggesting that EpCAM does not act in a strictly cell-autonomous manner, but is most likely also required in the neighboring cells to allow proper ruffle formation. Together, these data indicate that EpCAM is required for processes of epithelial morphogenesis driving the cell shape changes and spreading of the EVL during zebrafish epiboly.

### *epcam* mutant EVL cells display compromised basal protrusion formation, basally extended apical junctional complexes, and reduced E-cadherin levels in the basolateral membrane

**Figure 7:**
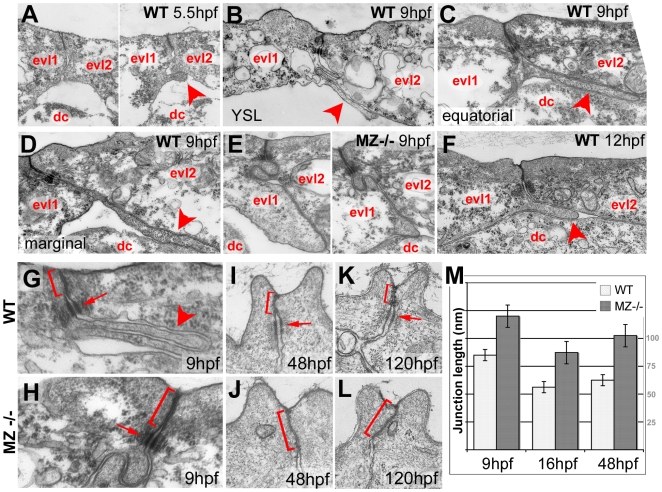
Basal protrusions and apical junctions are altered in MZ*epcam* mutants. (A–L) Transmission electron micrographs of EVL–EVL interphase region of wild-type (A–D,F–K) and MZ*epcam* mutant embryos (E,H–L) at indicated developmental stages. EVL and deep cells are indicated (evl1/2, dc; A–F), basal protrusions are marked by arrowheads (A–D,F,G), apical junctional complexes (AJCs) and their sizes by brackets (G–K), and desmosomes by arrows (G–I,K). (A–D) In wild-type embryos, basal protrusions start to form at early gastrula stages (A). Their lengths increase during gastrulation (B–D), and decrease after gastrulation is completed (F). Lengths in marginal EVL cells (D) are usually higher than in equatorially located EVL cells (C; see also Figure 6). Protrusions are also formed by marginal EVL cells spanning the cell-free zone between the deep cell layers and the yolk syncytial layer (YSL; [10]) (B). In mutants, basal protrusions are much shorter and broader (E). (G–L) AJCs are basally extended in the mutants (H,J,L), compared to wild-type siblings (G,I,K). Panel (M) shows graphical illustration of average lengths of AJCs in wild type and mutant cells at indicated developmental stages. n = 12; standard errors are indicated.

To further investigate the cellular basis of the epithelial defects of MZ*epcam* mutants, we performed immunohistochemistry and transmission electron microscopy (TEM). Consistent with the ruffles visualized via phalloidin stainings (see above; Figure 5I), TEM revealed protrusions at the basal side of wild-type EVL cells (Figure 7A–7D). These protrusions started to form during early gastrulation stages (Figure 7A), increased in length during gastrulation (Figure 7B–7D), and shortened again during segmentation stages (Figure 7F). They were in tight physical contact with the basal side of neighboring EVL cells, and even formed in most marginal EVL regions that are devoid of underlying deep cells (Figure 7B). In agreement with the altered actin pattern (Figure 5J), these basal protrusions were much broader and shorter in MZ*epcam* mutants (Figure 7E). TEM sections further revealed that the closely sealed apical junctional complexes (AJCs) of EVL cells were basally extended in MZ*epcam* mutants compared to wild-type controls (Figure 7G–7M). This difference was apparent throughout all investigated developmental stages from mid gastrulation through day 5 of development. However, desmosomes appeared morphologically unaltered (Figure 7G and 7H; and data not shown).

**Figure 8:**
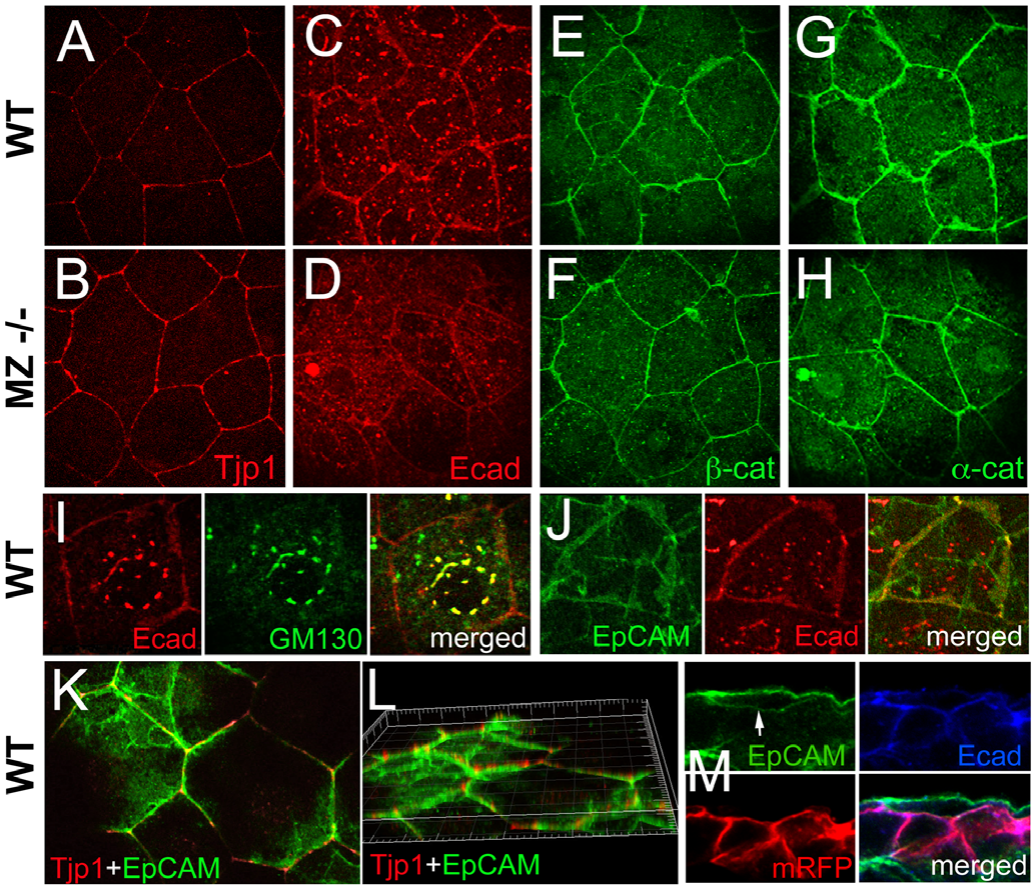
In wild-type EVL cells, EpCAM is localized at the basolateral membrane and excluded from the apical domain, while the molecular composition of apico-basal membranes is altered in *MZepcam* mutants. (A–H) Immunodetection of Tjp1 (A,B), Ecad (C,D), β-catenin (E,F) and α-catenin (G,H) in the EVL of wild-type (A,C,E,G) and MZ*epcam* mutant embryos (B,D,F,H) at 90% epiboly stage. Images were acquired and processed using identical settings for mutant and wild-type samples. Mutants display increased membranous levels of the tight junction protein, but reduced levels of the cadherin-catenin complex. (I–M) Localization studies in wild-type EVL cells at 90% epiboly stage. (I) Double immunodetection of Ecad and the Golgi marker GM130-GFP [80], single and merged channels, revealing perinuclear E-cadherin in the Golgi apparatus. (J) Double immunodetection of Ecad and EpCAM-GFP, single and merged channels, revealing co-localization in a salt-and-pepper-like pattern (compare with [62]). (K,L) Double immunodetection of Tjp1 and EpCAM-GFP, revealing that EpCAM-GFP is localized basal to Tjp1; (L) shows rotated Z-stack. (M) Triple immunodetection of EpCAM-GFP, Ecad and RFP (labeling transplanted deep cells lacking EpCAM-GFP); single and merged channels. The white arrow indicates EpCAM-GFP localized in the basal membrane of the EVL cell (the underlying RFP-positive deep cells lack EpCAM-GFP).

In addition, consistent with the basal extension of TJs, immunohistochemistry revealed an increase in the staining intensity for Tight junction protein 1 (Tjp1/ZO1) [61] in the lateral membranes of MZ*epcam* mutant EVL cells (Figure 8A and 8B). Supporting results were obtained in EpCAM gain-of-function studies in madine-darby canine kidney (MDCK) cells, which upon transfection with GFP-EpCAM displayed reduced membranous signals for the TJ components Tjp1 and Occludin, whereas desmoplakin stainings appeared unaltered (Figure S4). However, opposite alterations were observed for Ecad and its cytoplasmic binding partners α- and β-catenin (Figure 8C–8H), all of which displayed reduced membranous staining in MZ*epcam* mutant EVL cells. In addition, perinuclear Ecad staining in the Golgi apparatus (Figure 8I) was strongly reduced in mutant cells (Figure 8D) compared to wild-type controls (Figure 8C). Together, these data indicate that EpCAM promotes the presence of cadherin-catenin complexes in the basolateral domain of EVL cells, whereas it opposes the presence of TJ proteins. In line with these differential effects, EpCAM was co-localized with Ecad in lateral (Figure 8J) as well as basal membranes (Figure 8M) of wild-type EVL cells, whereas Tjp1 was localized apical of the EpCAM domain (Figure 8K and 8L), consistent with results obtained in cultured epithelial cells [62]. To investigate whether compromised basal protrusion formation in MZ*epcam* mutants might be caused by this gain of TJPs or loss of E-cadherin, we inactivated zebrafish *tjp1–3* [14],[61] via MO injection, and re-introduced E-cadherin by injecting different mRNAs or plasmid DNAs encoding mouse E-cadherin (see Materials and Methods). However, neither of the treatments led to a significant restoration of ruffle formation (Figure S5; and data not shown), suggesting that the molecular effects of EpCAM might be more complex.

### EpCAM and Ecad play redundant roles to promote cell–cell adhesiveness within the EVL of gastrulating embryos

**Figure 9:**
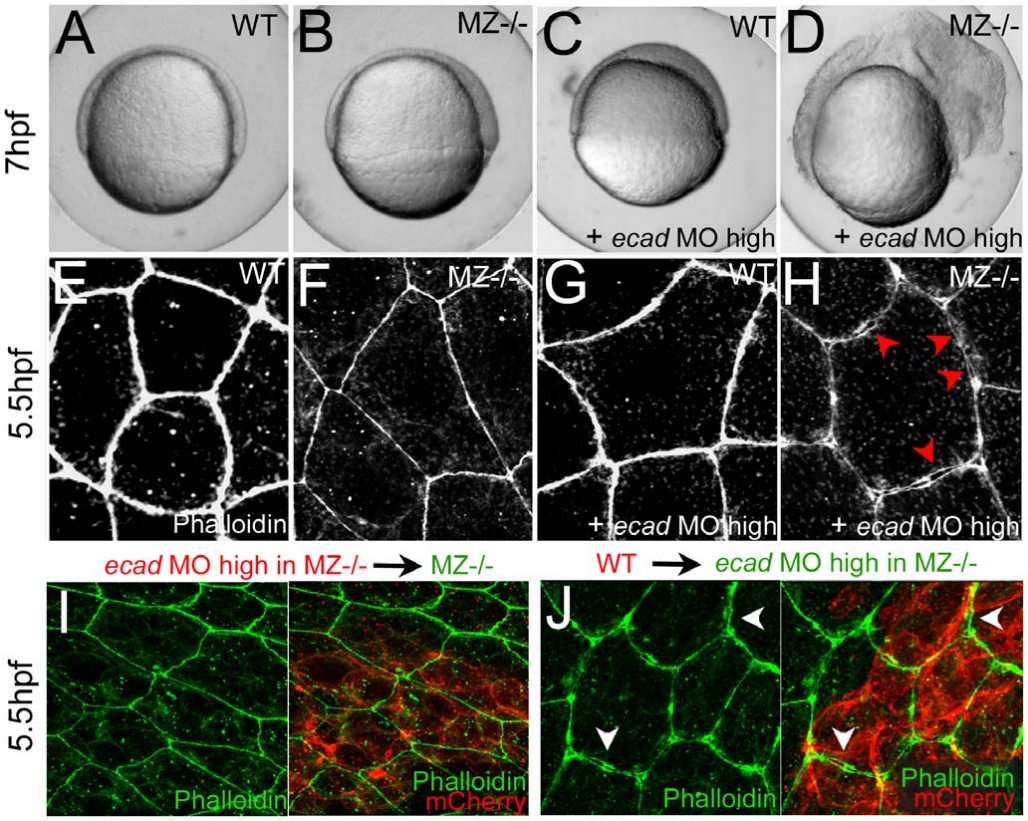
Combined complete loss of EpCAM and Ecad leads to compromised intercellular adhesion within the EVL of early gastrula embryos. (A–D) Live animals at the 70% epiboly stage (7 hpf); dorsal to the right; (E–J) phalloidin stainings of the actin cytoskeleton at 50% epiboly (5.5 hpf). (A,E) wild-type control (WT); (B,F,I) MZ*epcam* mutant control; (C,G) WT injected with high amounts of E-cadherin morpholino oligonucleotide (*ecad* MO); (D,H,J) MZ*epcam* mutant injected with high amounts of *ecad* MO. The regular *ecad* morphant (C) displays an arrest of deep cell epiboly at the equator, as previously described [10]–[12],[87], whereas EVL morphology appears normal (G). In contrast, EVL cells of the *ecad*;*epcam* double morphant/mutant embryo lose contact to each other (H; red arrowheads point to sites disassembly sites), and the embryo lyses (D). (I,J) The same EVL disassembly is observed in genetic mosaics, in which *ecad*;*epcam* double morphant/mutant EVL are positioned above transplanted wild-type deep cells (J; white arrows to disassembly sites), whereas wild-type EVL above *ecad*;*epcam* double morphant/mutant deep cells appear normal (I). Deep cells were transplanted at late blastula stages (3.5 hpf), and immunostained for the tracing marker mCherry in red.

Complementing the failed rescue experiments described above, we next analyzed whether the defects of MZ*epcam* mutants get enhanced upon concomitant loss of the (remaining) E-cadherin. For this purpose, different amounts of *ecad* MO were injected into MZ*epcam* mutant embryos. In contrast to *epcam*, *ecad* is expressed both in the EVL and in the deep cells. Strong *ecad* morphants and *ecad* null mutants display defects during epiboly movements of deep cells (see Introduction), whereas the EVL appeared normal (Figure 9C and 9G). In striking contrast, and unlike uninjected MZ*epcam* controls (Figure 9B and 9F), MZ*epcam* mutants injected with high amounts of ecad MO displayed severely compromised cell-cell adhesion of EVL cells at early gastrula stages (5.5 hpf; Figure 9H), leading to embryo lysis at mid gastrulation (Figure 9D). This effect was strictly layer-autonomous and independent of E-cadherin in the underlying deep layer, as indicated by the disrupted morphology of the EVL in genetic mosaics with a mutant EVL and wild-type deep layers (Figure 9J), but a wild-type morphology in the opposite combination (Figure 9I). This indicates that, while dispensable per se, EpCAM and Ecad together are required for intercellular adhesion within the EVL.

### EpCAM and Ecad from the EVL promote epiboly movements of underlying deep cells

**Figure 10:**
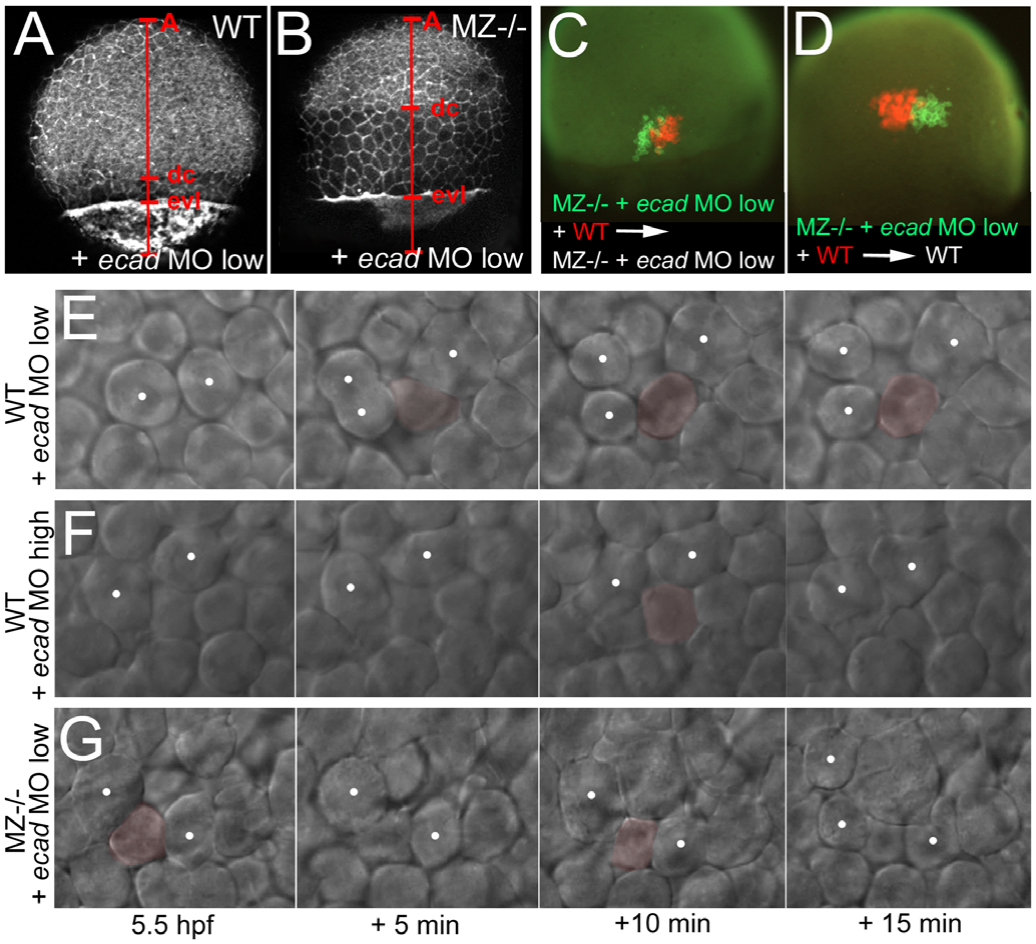
Concomitant partial loss of Ecad in the EVL of *MZepcam* mutants leads to severely compromised epiboly movements of deep cells. (A–D, E,G) wild-type (A,C,E) or MZ*epam* mutant (B,D,G) embryos injected with low amounts of *ecad* MO; (F) wild-type embryo injected with high amounts of *ecad* MO. (A,B) Phalloidin stainings of the actin cytoskeleton at 80% epiboly stage (for explanation of labeling, see Figure 5). The *ecad* hypo-morphant appears normal (A; compare with Figure 5C as un-injected control), whereas partial loss of Ecad activity in the *epcam* mutants leads to an arrest of deep cell epiboly (B; note the reduced A-dc distance compared to panel A and to Figure 5D). In contrast, EVL epiboly is not more affected than in the un-injected mutant (B; note the normal A-evl distance compared to Figure 5D). (C,D) Genetic mosaics at 80% epiboly stage, after co-transplantation of deep cells from wild-type embryos (anti-RFP immunostaining; in red) and from MZ*epcam* mutants injected with low amounts of *ecad* MO (anti-GFP immunostaining; in red), shortly before the onset of epiboly (4 hpf). Epiboly behavior of the transplanted deep cells does not depend on their own genotype, but on that of the host embryo/the overlying EVL. (E–G) Stills of time-lapse video recordings of the external layer of deep cells, starting at early gastrula stages (5.5 hpf). For evaluation, only cells were considered that moved up from internal into the external layer of the deep cells during the first half (0–10 min) of the recordings. Individual cells are pseudo-colored in light red when in the external layer, but un-colored when in lower layers. Note that the cell in (E) remains in the external layer throughout the second half of the video (10–20 min), whereas cells in (F,G) move up and down. White spots label representative neighboring cells of the external deep cell layer, demonstrating their increasing distance in (E), but constant distances in (F,G).

As described above, EpCAM and Ecad are not only co-localized at the lateral, but also at the basal membranes of EVL cells, facing the underlying deep cells (Figure 8M). Previous studies have shown that Ecad is primarily required to mediate the anchorage of deep cells to the inner surface of the EVL during radial intercalation processes driving the epiboly of deep cells (see Introduction). To test whether EpCAM from EVL cells might also be involved in this anchorage, we carried out genetic interaction studies, injecting ineffectively low amounts of *ecad* MO into wild-type or MZ*epcam* mutant embryos. In mid gastrula wild-type embryos (80% epiboly stage) injected with such low amounts of *ecad* MO, epiboly of deep cells was normal (Figure 10A; n = 25/25; compare with Figure 5C). In contrast, and unlike the very moderate deep cell epiboly defects of un-injected MZ*epcam* mutants (Figure 5D), mutants injected with the same low amounts of *ecad* MO displayed an arrest of deep cell epiboly at the equator of the embryo (Figure 10B; n = 27/29), similar to the defects of embryos injected with highest amounts of *ecad* MO (data not shown; n = 20/21) [10]. Time-lapse recording at early gastrulation stages further revealed crucial differences in the behavior of deep cells. In un-injected embryos or in wild-type embryos injected with low amounts of *ecad* MO, radially intercalating deep cells remained in the exterior layer of the deep layers, most likely stably attached to the inner surface of the EVL (Figure 10E and data not shown; n = 6/6; 3 videos), whereas in MZ*epcam* mutants injected with low amounts of *ecad* MO, cells usually moved back into more interior layers of the deep layers (Figure 10F; n = 4/5; 3 videos), similar to the situation after complete knock-down of *ecad* (Figure 10G; n = 5/5; 2 videos). Interestingly, this effect on deep cell behavior again seemed to primarily depend on EpCAM and Ecad function within the overlying EVL. Thus, in genetic mosaics, deep MZ*epcam* mutant cells injected with low amounts of *ecad* MO displayed the same epiboly behavior like their wild-type neighbors, which was compromised when the host and the EVL were mutant (Figure 10C; n = 5/5), but normal when the host and the EVL were wild-type (Figure 10D; n = 6/6). Together, this suggests that EpCAM from the EVL supports the function of Ecad to drive epiboly of deep cells.

## Discussion

EpCAM is a well-established carcinoma marker that in addition to its diagnostic value for rapidly growing tumors of epithelial origin is used as a potential target for immunotherapy [17],[29],[63],[64]. Most of the current studies dedicated to the function of EpCAM have used in vitro systems, revealing a rather pleiotropic nature of EpCAM's roles during tumor progression and normal epithelial development (reviewed in [18],[31]). Initially proposed to be a cell-cell adhesion molecule [23],[33],[35], more recent studies have pointed to additional and, to some extent, seemingly contrary roles of EpCAM in diverse processes such as signaling, cell migration, proliferation and differentiation. Some of these data have led to the notion of a “double-face” of EpCAM function in promoting both adhesion/tissue integrity and cell motility/morphogenesis/metastasis [18],[31]. Here, using zebrafish genetics, we present in vivo loss-of-function data largely in line with this notion, and discuss how the double-face effect might be achieved on the cellular level.

### The zebrafish EpCAM mutants and morphants

Apart from zebrafish, no EpCAM mutants have been described in any other organism. Two mutant zebrafish alleles were isolated, both of which are most likely EpCAM nulls (Figure 1 and Figure 2). Maternal-zygotic (MZ) mutants lacking both maternally and zygotically supplied *epcam* gene products display early epiboly and later skin defects, whereas epiboly seems to be normal in zygotic mutants and *epcam* morphants. The weaker phenotype of zygotic mutants suggests that maternally supplied *epcam* gene products, either mRNA or protein, are sufficient to drive epiboly. The lack of epiboly defects in *epcam* morphants further points to maternal EpCAM protein. Translational start site morpholinos as used here target both maternally and zygotically provided mRNA, but not maternal protein, suggesting that the weaker phenotype of *epcam* morphants compared to MZ*epcam* mutants is due to the presence of maternally provided EpCAM protein. EpCAM-specific antibodies will be required to test this notion.

In addition to the epiboly and skin defects that are the focus of this work, *epcam* zebrafish mutants display compromised otolith formation in the developing inner ears (Figure 1 and [45]). The cellular and molecular basis of this phenotype is unclear, however, similar otolith defects have been described for zebrafish mutants in the tight junction component Claudinj [65]. Another site of prominent zygotic zebrafish *epcam* expression are the neuromasts of the lateral line (Figure 2). According to a previous report, loss of EpCAM function by morpholino injection leads to defects in neuromast deposition during the posterior-wards migration of the lateral line primordium [53]. Although nicely in line with the concept of a role of EpCAM during epithelial morphogenesis, our analysis of MZ*epcam* mutants could not confirm this phenotypic trait (Figure S6). Indeed, Villablanca et al. had to inject highest amounts of morpholinos to obtain the phenotype, suggesting that it might have been an unspecific effect.

### The role of EpCAM in the enveloping cell layer of gastrulating zebrafish embryos

**Figure 11:**
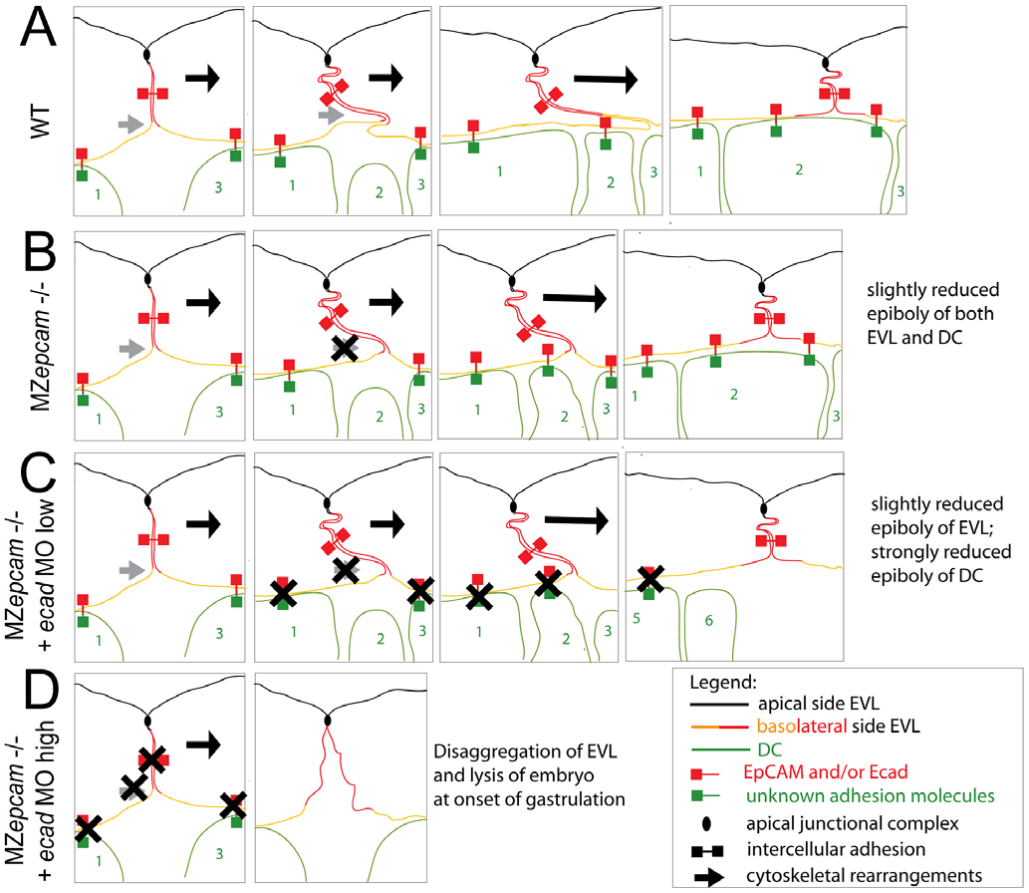
Model of cellular processes at play during EVL epiboly in wild-type embryos and different EpCAM/Ecad-deficient combinations. Schematic drawings of EVL–EVL–deep cell interphase regions at, from left to right, progressing stages of zebrafish gastrulation. Left panels represent pre- or early gastrula stages, middle panels mid gastrula stages, and right panels post gastrula stages. (A) wild-type conditions; (B) MZ*epcam* mutant; (C) MZ*ecpam* mutant with concomitant partial loss of E-cadherin; (D) MZ*epcam* mutant with concomitant complete loss of E-cadherin. At the onset of gastrulation, EVL cells start to from basal protrusions, initiated by cytoskeletal rearrangement to overcome the cortical surface pressure, as indicated by grey arrow. Protrusions attach to the basal surface of adjacent EVL cell and increase in length. This leads to a progressive narrowing of the lateral side of the EVL cell, and to an increase of its basal surface, and eventually of its entire horizontal size. Protrusion growth is dependent on EpCAM function, which might be involved both in the cytoskeletal rearrangement (crossed out grey arrow in B), and/or in mediating adhesion between the protrusion and the adjacent cell. The latter notion, which would be in line with the existence with homophilic EpCAM trans bonds, is supported by results obtained via re-introduction of EpCAM into single EVL cells (Figure 6). The gain of basal surface and the transition from a curved to a straighter shape increases the chance of successful attachment of emerging deep cells (green cell number 2) to the inner surface of the EVL, thereby linking progression of EVL and deep layer epiboly. However, simultaneous further movement forces are required, such as directed pulling from the EVL margin, to account for a net gain of basal surface throughout the entire layer (indicated by black arrows). Otherwise, the surface gain of one cell would be equalized by a corresponding loss of free surface in the adjacent cell. These dynamic EVL cell shape changes are layer-autonomous and largely independent of the underlying deep cells. Otherwise, it would be impossible to uncouple EVL from deep layer epiboly, as seen in strong *ecad* mutants and morphants [10]. In contrast, deep layer epiboly is strictly dependent on a functional EVL. Partial loss of Ecad in MZ*epcam* mutant EVL cells causes destabilization of the adhesion between the basal side of EVL cells and underlying deep cells (C). As a consequence, deep cells can move back into more internal layers, and deep layer epiboly is severely compromised. In contrast, EVL epiboly is only slightly retarded, as in regular MZ*epcam* mutants (due to the persistence of the other moving forces indicated by the black arrow). Adhesion among EVL cells becomes only severely compromised upon combined complete loss of EpCAM and Ecad (D). This indicates that EVL–deep cell adhesion requires higher Ecad/EpCAM levels than EVL–EVL cell adhesion (see Text S1 for further details). Our chimera analyses further indicate that for proper deep cell epiboly, both EpCAM and Ecad are only crucial in the EVL, but dispensable in the deep cells themselves, suggesting that here, other or additional adhesion molecules (indicated in green) might be involved in mediating trans-adhesion to the overlying EVL. It remains unclear whether EpCAM and Ecad act in a complex, or as rather independent molecules with partially redundant adhesive properties. Therefore, this model does distinguish between the two molecules (commonly indicated in red). Not considered is the basal extension of apical junctional complexes in mutants.

During gastrulation, *epcam* is exclusively expressed in cells of the enveloping layer (EVL; see Introduction). Nevertheless, MZ*epcam* mutants display moderate defects during epiboly movements of both the EVL and the underlying deep cell layers (Figure 5). Our chimeric analyses in combination with E-cadherin inactivation indicate that the deep cell defects are secondary consequences of failed EpCAM function in the EVL, whereas the EVL defects themselves are layer autonomous (see below). During their spreading over the yolk, EVL cells normally change their shape and flatten out along the radial axis to increase their horizontal size. The cellular mechanisms underlying this transition are largely unknown. Here, we show that they involve basal protrusive activity of EVL cells, and that this activity is severely compromised in MZ*epcam* mutants, leading to an overall reduction in the average horizontal size of mutant EVL cells (Figure 5). Recently, a similar impairment of EVL epiboly has been described for MZ*pou5f1* mutants. However, in this case, epiboly defects are accompanied by increased, rather than reduced protrusive activity of EVL cells [66]. This indicates that both gain and loss of protrusive activity can compromise epiboly. Future experiments have to address the functional connection between *pou5fl* and *epcam*. In MZ*epcam* mutants, the lack of basal activity is accompanied by a basal extension of apical junctional complexes (AJCs) (Figure 7), and by increased levels of TJ components in apico-basal membranes, whereas levels of E-cadherin (Ecad) and catenins are reduced (Figure 8). These effects are consistent with the co-localization of EpCAM and Ecad in the basolateral membranes of wild-type EVL cells, and their exclusion from the apical Tjp1 domain (Figure 8). Together, this suggests that EpCAM pushes the molecular composition of apico-basal membranes from TJ towards AJ components. In addition to the membrane, mutants displayed reduced perinuclear Ecad staining in the Golgi apparatus, suggesting that EpCAM also affects de novo synthesized Ecad protein. Currently, we cannot distinguish which of the observed phenotypic traits are primary, and which secondary. However, neither knockdown of tjp1–3 nor re-introduction of Ecad led to an alleviation of the mutant phenotype or a restoration of basal protrusions, suggesting that EpCAM might have multiple targets and interaction partners, rather than acting via a single mediator. Generally, protrusion formation is driven by rearrangements of the cortical cytoskeleton that are coordinated with local modulations in cellular adhesiveness [67]. Interestingly, according to our TEM studies, the protrusions of EVL cells primarily attach to the basal membrane of adjacent other EVL cells, rather than to underlying deep cells or extracellular matrix components, while protrusions remain much shorter and broader in MZ*epcam* mutants (Figure 7). In this light, it is tempting to speculate that the thinning of EVL cells underlying EVL epiboly might be driven by the “crawling” of basal protrusions on the basal surface of adjacent EVL cells, and that EpCAM might be particularly involved in modulating cell-cell adhesiveness and cortical tension in basolateral domains of EVL cells (Figure 11A and 11B).

### In vivo interaction of EpCAM and E-cadherin to regulate layer-intrinsic and trans-layer adhesion of the zebrafish EVL

In contrast to cell culture studies identifying EpCAM as a functional antagonist of Ecad [20],[35], we found that zebrafish EpCAM and Ecad tightly interact and enhance each other's effects to promote EVL integrity as well as deep cell epiboly. While each of them per se was dispensable for EVL integrity, combined loss of both EpCAM and Ecad led to severe and layer-autonomous EVL disassembly during early gastrulation stages (Figure 9). This indicates that EpCAM and Ecad play essential, yet redundant roles to mediate proper cell-cell adhesion among EVL cells (Figure 11D). Similarly, partial inactivation of Ecad, which had no effect in a wild-type background, led to a complete arrest of deep cell epiboly in MZ*epcam* mutants (Figure 10A and 10B). Interestingly, also here, the effect depended purely on EpCAM and Ecad function in the EVL (Figure 10C and 10D). On the cellular level, the epiboly arrest was accompanied by a failure of intercalating deep cells to remain in the external deep layer directly underneath the EVL (Figure 10E–10G), pointing to defects in the adhesion between EVL and underlying deep cells, thus, across different layers (Figure 11C) [12]. In this light, both the tissue integrity defects caused by loss of EpCAM function in the background of complete *ecad* inactivation, and the tissue morphogenesis defects caused by loss of EpCAM function in the background of partial *ecad* inactivation, seem to result from reduced intercellular adhesion. But what are the reasons for the exclusive effects on epiboly, but not EVL-EVL adhesion, in the *ecad* hypomorphic background? Differential contributions of Ecad and EpCAM to EVL-EVL versus EVL-deep cell binding could be one factor. EVL-EVL cell adhesion involves apical junctional complexes and desmosomes, and might therefore be less dependent on “free” basolaterally localized EpCAM and Ecad than EVL-deep cell adhesion, which lacks such junctions. Alternatively or in addition, the deep cell epiboly arrest might be due to combined effects on cell adhesiveness and its dynamic regulation. During gastrulation, deep cells undergo massive spatial rearrangements in addition to radial intercalations, one of the driving forces of epiboly. For instance, they simultaneously move from ventrolateral into dorsal regions of the embryo to form the embryonic body axis [67]. Therefore, neighborships between EVL and deep cells need to change rather rapidly. In this light, it is feasible to speculate that EpCAM might also be involved in regulating the dynamic dis- and re-assembly of EVL-deep cell contacts to allow proper morphogenesis. Cadherin contacts can be regulated at multiple levels [68], and membrane localization of Ecad has recently been shown to be dynamically regulated via endocytosis and recycling during gastrulation movements of deep cells in zebrafish and frog embryos [67],[69],[70]. Furthermore, the cytoplasmic domain of EpCAM contains an NPXY internalization motif [19], which could possibly trigger the concomitant endocytosis of Ecad, comparable to the recently revealed role of the transmembrane protein FLRT3 in Xenopus embryos [70].

In sum, we propose that EpCAM and Ecad play rather similar, and partially redundant roles during zebrafish gastrulation. Furthermore, they seem to mutually influence each other, with a positive effect of EpCAM on membranous Ecad levels and, possibly, Ecad synthesis and recycling. Subject to dynamic regulation, cadherins are well known for their multiple, and seemingly contrary effects, not only promoting cell-cell adhesiveness and tissue integrity, but also cellular plasticity and cellular “grip” during morphogenesis. Similar mechanisms might underlie EpCAM's reported “double face” function also revealed in this work. Future biochemical studies will be necessary to elucidate the molecular basis of the EpCAM – E-cadherin partnership. Since they play largely redundant roles, they do not necessarily have to physically interact at all. They could for instance mediate different modes of adhesion, cadherins in connection with the highly structured actin cytoskeleton and EpCAM more independently of the cytoskeleton [18],[34]. Consistent with this notion, our immunolocalization studies indicate a salt-and-pepper like distribution of EpCAM and Ecad, rather than complete co-localization in the basolateral membrane of EVL cells (Figure 8J). However, it is interesting to note that in the context of nuclear signaling, complex formation between the intracellular domain of EpCAM and β-catenin has been observed [41]. Furthermore, more indirect mechanisms might be at play, involving adapter or signaling proteins [71].

### The later role of EpCAM in the skin

Similar effects of EpCAM might also account for the later defects in the mutant skin. From mid-segmentation onwards (14 hpf), the outer EVL, now also called periderm, is juxtaposed against a single layer of basal keratinocytes, which derive from the ventral ectoderm, a subpopulation of deep cells initially located in ventral-animal regions of the pregastrula embryo [5]. Both layers display *epcam* expression, and both display compromised epithelial integrity in *epcam* mutants. However, the defects in the periderm are already apparent when the basal layer is still normal (16 hpf; Figure 3C–3E). This later periderm defects are very similar to those caused by combined loss of EpCAM and E-cadherin during gastrulation. It remains unclear, however, why in contrast to the early stages and despite its maintained expression, E-cadherin fails to compensate for loss of EpCAM after skin formation. Furthermore, consistent with the aforementioned effect of EpCAM from the EVL on epiboly movements of the underlying deep cells, our chimera analyses reveal that defects in the basal layer are due to a non-autonomous effect from the EVL (Figure 3H and 3I). Also, similar to the situation during epiboly, *epcam* mutants display a persistent basal extension of AJCs in EVL cells during such later stages (Figure 7J and 7L). Although the role of tight junctions in cell adhesion is still disputable, it is generally accepted that their impact compared to adherence junctions and desmosomes is minor [72],[73]. In this light, and in light of the observed negative effect of the MZ*epcam* mutation on membranous Ecad levels and cell-cell adhesiveness during gastrulation, we assume that the epithelial defects of the mutant periderm are due to reduced, rather than enhanced cell-cell adhesiveness. In addition, defects might be enhanced by reduced epithelial plasticity. Consistent with this notion, initial epithelial lesions in the periderm of *epcam* mutants were most prominent on the tailbud (Figure 3B) and the head, regions that undergo massive morphogenesis and that are exposed to highest mechanical stress. Very similar shedding of skin cells was previously described for *hai1* mutants, which display partial epithelial-mesenchymal transitions of basal keratinocytes [56]. However, no mesenchymal-like behavior was observed in time-lapse recordings of basal keratinocytes or EVL cells of *epcam* mutants (K.S. and M.H., unpublished observations). In sum, these data suggest that similar to the defects during epiboly, the later skin defects of *epcam* mutants are due to compromised intercellular adhesion and cellular plasticity of epithelial cells.

In light of the reported roles of EpCAM in multiple other cellular processes, such as cell proliferation and cell differentiation, we also investigated whether and when skin cells of MZ*epcam* mutants develop corresponding defects. However, in contrast to other reported zebrafish skin mutants [74], EVL and basal cells showed normal levels of terminal differentiation markers (Keratin, ATPases; Figure S7). Also, although EVL and basal cells displayed an up to 50% increase in proliferation, this hyper-proliferation only became apparent several hours after the epithelial defects (Figure 4B). Furthermore, aggregates of both EVL and basal cells displayed similar rates of BrdU incorporation like regions remote of the aggregates (Figure 4C). An interesting side outcome of the BrdU incorporation studies, also confirmed by time-lapse recordings (K.S. and M.H., unpublished data), was the demonstration that EVL cells divide at all. This had not been shown before. Rather, it was widely believed that the EVL grows by cell shape changes, and that it later sloughs off, being replaced by cells deriving from basal cells [5]. However, our data suggest that the growth of the two larval zebrafish skin layers might involve layer-autonomous, horizontal cell divisions, rather than or in addition to vertical growth/replacement, the typical concept of stratified epithelia.

In conclusion, our studies indicate that loss of EpCAM in the developing zebrafish skin primarily leads to compromised epithelial plasticity and adhesiveness, whereas hyper-proliferation is a secondary consequence, possibly due to the loss of contact inhibition. Similarly, the higher susceptibility to bacterial infections and enhanced inflammation of *epcam* mutants (Figure S2, Video S1, Video S2) are most likely secondary consequences of compromised skin integrity.

In contrast to embryonic and larval stages, however, we found EpCAM to be largely dispensable after metamorphosis, when the skin has become multi-layered. The reason for this later dispensability remains unclear. Functional redundancy with other genes could be one explanation. In mammals, an EpCAM homologue called Trop2/Tasctd2 exists, which shows approximately 50% sequence identity to EpCAM, and which has most likely evolved via a retrotranspositional event [75]. In contrast, searches of zebrafish databases failed to identify further EpCAM-related sequences [53] (M.H. and K.S., unpublished data), suggesting that zebrafish *epcam* is a single gene. Interestingly, adult zebrafish *epcam* mutants even displayed normal cutaneous wound healing (K. S. and M.H., unpublished data), indicating that despite its reported elevated expression during epithelial regeneration of the mammalian liver and kidney [24],[28], EpCAM is not required for epithelial morphogenesis that takes place during zebrafish skin repair. Future experiments have to reveal whether *epcam* mutants are less susceptible to epithelial tumor formation, which would reinforce its suitability as a target for anti-carcinoma therapies.

## Materials and Methods

### Fish husbandry

The EpCAM alleles *hi2836* and *hi2151* were isolated during an insertional mutagenesis screen [76]. Unless noted otherwise, the *hi2151* allele was used. *hi2151* mutants were obtained from heterozygous (Z+/−) or homozygous parents (MZ−/−). The *Tg(krt4: egfp)gz7* and *Tg*(*βactin:hras-egfp*) (allele vu119) transgenic lines have been described previously [55],[77].

### Cloning of cDNAs and RNA synthesis and microinjection

The full-length coding region of zebrafish EpCAM cDNA (GenBank accession number NM_213175) was amplified via RT-PCR and cloned into pCRII-TOPO (Invitrogen) or upstream of eGFP into pCS2+ plasmid [78]. To generate pCS2-Ecad-HA, the full-length coding region of mouse E-cadherin was amplified and cloned upstream of HA into pCS2+. For antisense probe synthesis, pCRII-EpCAM was linearized with XbaI and transcribed with SP6 RNA polymerase. For sense RNA synthesis for microinjection, pCS2-EpCAM-eGFP, pCS2-Ecad-HA and pCS2-mCherry (with farnesylation signal; generous gift from Erez Raz) were linearized with NotI and transcribed using SP6 Message Machine kit (Ambion); mGFP mRNA [79] and GM130-GFP mRNA were generated as described [80]. For rescue experiments, EpCAM-eGFP or Ecad-HA mRNA were injected at 150, 350 (EpCAM) or 100 µg/ml (Ecad), respectively (1.5 nl per embryo), pCS2-EpCAM-eGFP, pCDNA3.1-Ecad-GFP [81], pCS2-Ecad-HA, or pL31NU-Ecad-Venus DNA [82] at 350 µg/ml (EpCAM) or 100 µg/ml (Ecad), respectively.

### Tissue labeling procedures

In situ hybridizations were performed as previously described [83], using probes for EpCAM and *leukocyte-specific-plastin (lcp1)* [59]. Whole-mount immunostaining was carried out as described [83], using fluorescently-labeled secondary antibodies or the Vectastain ABC kit (Axxora) for enzymatic detection. Antibodies, dilutions used and sources were as follows: anti-p63 (1∶200, Santa Cruz), anti-GFP (1∶400, Invitrogen), anti-RFP (1∶200; antibodies-online GmbH, ABIN132020), anti-E-cadherin (1∶200, BD Biosciences), anti-ZO-1 (1∶200, Zymed), Alexa-Fluor-546 goat anti-mouse (1∶400, Invitrogen) and Alexa-Fluor-488 goat anti-rabbit (1∶400, Invitrogen). Tested anti EpCAM antibodies: A-20 (Santa Cruz), 1144 (Epitomics), HO-3 [84] and C-215 [85]. For paraffin sectioning (8 or 16 µm), stained embryos were dehydrated in ethanol series and clearing in toluene, the specimens were infiltrated with paraffin, embedded, and sectioned. Epidermal cell proliferation was assessed by BrdU incorporation followed by combined anti-p63 and anti-BrdU immunostaining as described [56]. Apoptotic cells were visualized by TUNEL staining using in-situ cell death detection kit (Roche). Phalloidin stainings of cortical actin cytokeleton were carried out with Alexa 488 or Alexa 594 Phalloidin (1∶200, Molecular Probes), as described [10].

### Morpholino oligonucleotides (MOs) injections and blebbistatin treatment

*epcam* MO (5′- GTGCAGAGACTTTCCGGCCATATTT-3′) was obtained from Gene Tools (Philomath, OR) and diluted in Danieau's buffer [86]. 1.5 nl of a 200 µM MO solution were injected per embryo at the 1 cell stage. The *ecad* MO was as described [87]. For complete knock-down, 1.5 nl of a 200 µM solution was injected, for synergistic enhancement studies, 1.5 nl of a 30 µM solution. The *tjp1*, *tjp2 and tjp3* MOs (generous gifts from Matthias Köppen and Carl-Philipp Heisenberg; [14]) were injected alone or together at 50 to 200 µM each); the *msn1* MO was as described [10] and injected at 200 µM.

### Cell transplantations

Clusters of mGFP-labeled basal keratinocytes were obtained by homotopic transplantation of approximately 50 non-neural ectodermal cells from Tg(*βactin:hras-egfp*) transgenic donor embryos into non-transgenic hosts at 6 hpf. Recipients were fixed at 36 hpf, subjected to anti-GFP and anti-p63 immuno-fluorescence staining, mounted in 1.5% low melting agarose and analyzed by confocal microscopy. For localization studies of EpCAM-eGFP on the basal side of EVL cells (Figure 8), deep cells of *mCherry* mRNA-injected cells were transplanted at the sphere stage into *EpCAM-eGFP* mRNA-injected hosts. Embryos were fixed at the 90% epiboly stage for anti-RFP and anti-GFP immunohistochemistry, sectioned, and analyzed via confocal microscopy. To distinguish whether for proper EVL adhesion, EpCAM and Ecad are required in the EVL cells themselves or in underlying deep cells (Figure 9), chimeric embryos were generated by transplanting deep cells at the sphere stage from mCherry-labelled wild-type or MZ*epcam* donors into unlabelled MZ*epcam* hosts injected with high amounts of *ecad* MO, or vice versa, followed by fixation at the 90% epiboly stage for anti-Cherry and phalloidin staining. To investigate whether for deep cell epiboly, Epcam and Ecad are required in the deep cells or in the EVL (Figure 10), cells from *mCherry* mRNA-injected wild-type or MZ*epcam* mutant donors were transplanted next to cells from *mGFP* mRNA + low *ecad* MO-injected MZ*epcam* mutant donors into unlabelled MZ*epcam* hosts or MZ*epcam* hosts injected with low *ecad* MO. Embryos were fixed at the 90% epiboly stage, and processed via anti-RFP and anti-GFP immunohistochemistry.

### Time-lapse videos and microscopy

For time-lapse in vivo imaging, embryos were mounted and recorded at a Zeiss Axiophot with Nomarski optics and a Hamamadzu Orca camera, as previously described [79]. 20-minutes videos were taken at lateral marginal regions, starting at shield stage (5.5 hpf), with 30 sec intervals. For Figure 10, single images form the time-lapse video recordings were imported into Adobe Photoshop, and single cells were pseudo-colored to aid the presentation [11].

Fluorescent images were taken with a Zeiss Confocal microscope (LSM510 META); Transmission light microscopy was performed on a Zeiss Axiophot or Leica MZ-8 stereomicroscope. For transmission electron microscopy, wild-type and mutant fish were fixed with 2.5% glutaraldehyde in PBS for 30 minutes each at ambient temperature and then on ice. After washing with PBS, the larvae were post-fixed with 1% osmium tetroxide in 100 mM phosphate buffer pH 7.2 for 1 hour on ice, washed with H_2_0, stained with 1% aqueous uranylacetate for 1 hour, dehydrated in a graded series of ethanol and finally embedded in Epon. Ultrathin sections were stained with uranyl acetate and lead citrate and viewed in Philips CM10 electron microscope.

## Supporting Information

Figure S1 *epcam* mutants form skin cell aggregates. (A) Confocal image (merged Z-stack) of basal keratinocytes of wild type control (WT) and maternal/zygotic *epcam* mutant (MZ−/−) embryos at 2 days post fertilization (dpf), after anti-p63 immunostaining of basal keratinocytes. Basal cell aggregates of mutant are indicated by red arrows. (B,C) Overviews (upper panels) and magnified views of tail region (lower panels) of live WT (left panels) and MZ−/− mutants (right panels) at 4 days post fertilization (dpf) (B) and 21 dpf, when the skin becomes multi-layered (C). The epidermal aggregates of mutants persist during further larval development (B; indicated by red arrows), whereas they are less prominent at 21 dpf (C). In addition, otoliths have recovered and acquired normal size at 21 dpf (C; indicated by black arrows). However, the skin of the mutant has a rougher morphology, the mutant is of reduced size (C; see different lengths of scale bars = 1 mm), and fin development is delayed, as judged by the less advanced fin ray formation in the developing unpaired anal, dorsal and tail fins (C; indicated by black arrowheads in wild-type animal). Further analyses have to reveal whether this is a skin/fin-specific defect, or a consequence of generally delayed development, growth and metamorphosis.
(3.16 MB TIF)

Figure S2 MZ*epcam* mutants display increased susceptibility to cutaneous infections. All panels show lateral views on the tail region of embryos at 2 dpf, after whole mount in-situ hybridization for *leukocyte-specific-plastin (lcp1)* mRNA, a marker for leukocytes (macrophages and neutrophiles) [59]. (A,B) In wild-type (WT; A) and maternal-zygotic *epcam* mutants (MZ−/−; B) kept in semi sterile medium, leukocytes are found mainly in the blood vessels. (C,D) Addition of *E. coli* to the incubation medium stimulates the presence of innate immune cells in the skin (indicated by blue arrows in wild-type fish; C). Skin inflammation is much stronger in the challenged mutant (D) than in the wild-type control (C), suggesting that the mutant is more susceptible to cutaneous infections.
(1.33 MB TIF)

Figure S3 In contrast to loss of *msn1* or treatment with blebbistatin, loss of *epcam* does not compromise the constriction of marginal EVL cells. (A–F) Fluorescent confocal images of phalloidin stained maternal zygotic-EpCAM mutant (MZ−/−) and wild type (WT) embryos at 70% epiboly. The leading EVL cells of both, mutant and WT embryos are constricted by an actin-myosin string as indicated by the presence of cells with shorter leading than trailing sides (A,B). This is in contrary to the release of constriction caused by in wild-type embryos upon treatment with 15 ug/ml blebbistatin (C) or injection of *msn1* MO (D) [10], suggesting that the EVL epiboly defects of MZ*epcam* and *msn1* mutants have a different cellular basis, and that the phenotype of MZ*epcam* mutants is not caused by reduced actin-myosin constriction at the EVL-YSL interface. Consistent with this notion, injection of msn1 MO or blebbistatin treatment in MZ*epcam* mutants, while releasing marginal constrictions (E,F), failed to synergistically enhance the EVL epiboly defects, but rather had pure additive effects (data not shown). Another possibility would have been that the epiboly defects of MZ*epcam* mutants are due to increased/precocious, rather than reduced actin-myosin constriction at the EVL-YSL interface. However, applying increasingly lower amounts of msn1 MO or blebbistatin to MZ*epcam* mutants, we never obtained an alleviation of the EVL epiboly defects (judged by the extrusion of the vegetal-most part of the yolk at late gastrula stages; compare with Figure 5B), also making this possibility very unlikely.
(1.59 MB TIF)

Figure S4 Overexpression of EpCAM in MDCK cells causes reduced levels of tight junction proteins. MDCK cells were transfected with expression vectors driving either GFP (A–C, G–I, M–O) or zebrafish EpCAM-GFP expression (D–F, J–L, P–R), plated on cover slips after FACS sorting one day after transfection, and stained for cell-cell junctional markers 48 hours after plating. Processed confocal images are shown as merges of entire Z-stacks. Transfectants were detected by GFP (B, H, N) or EpCAM-GFP expression (E, K, Q). Protein localization was analyzed with antibodies against Tjp1/ZO-1 (C, F), Occludin (Occ) (I, L), or Desmoplakin 1 and 2 (DPI/II) (O, R); panels (A,D,G,J,M,P) show overlays with GFP fluorescence. DAPI-stained nuclei are shown in blue (A,D,G,J,M,P); scale bar, 10 µm. Fluorescent labeling of key components of tight junctions reveals a reduction in membrane localization of Tjp1 in EpCAM- (F) versus GFP-transfected (C) cells. Similarly, Occludin staining is reduced in EpCAM expressing MDCK cells (I, L). However, proteins localizing to adherens junctions or desmosomes are not altered in expression or cellular distribution, as shown for DPI/II (O, R). This indicates that EpCAM expression in MDCK cells leads to a modification in apical junction complex assembly, resulting in a reduction of key tight junction proteins at the plasma membrane. (S) Immunoblot of lysats from FACS-sorted MDCK cells transfected with EpCAM-GFP (right lane) or, as control, GFP (left lane). EpCAM-GFP and GFP are expressed at comparable levels (upper panel). Expression of EpCAM-GFP leads to a significant in Tjp1/ZO1 protein levels (middle panel; arrow indicate full-length Tjp1/ZO1 protein). Gapdh was used as loading control (lower panel).
(14.76 MB TIF)

Figure S5 Knockdown of tight junction proteins 1–3 fails to restore ruffle formation in *epcam* mutants. Fluorescent confocal images of phalloidin stained wild type (WT) and maternal-zygotic EpCAM mutant (MZ−/−) embryos. Single and collective knock down of tight junction proteins 1–3 via antisense morpholino oligonucleotides (MO) injections into MZ−/− embryo (panel 2) failed to restore the protrusive activity of the mutant EVL cells back to the WT levels (panel 1, red arrows point to ruffles). If at all, rescue was very minor (orange arrow in panel 3 points to one of the few and rather small ruffles found in 12 investigated tjp1–3 MO-injected embryos).
(3.63 MB TIF)

Figure S6 Maternal-zygotic *epcam* mutants have unaltered numbers of deposited primary neuromasts. (A) 5 dpf old maternal-zygotic *epcam* mutants (MZ−/− ) and wild-type (WT) controls at 120 hpf, stained with 4-Di-2-ASP for vital labeling of differentiated hair cells in the larval lateral line. The red arrowheads point the deposited primary neuromasts. (B) Numbers of deposited primary neuromasts in WT control and MZ−/− mutants embryos at 2 dpf. Neuromasts were stained by their endogenous alkaline phosphatase activity [80]. Contrary to published data obtained from *epcam* morpholino studies [53], we could not detect significant differences in the numbers of deposited neuromasts between mutant and wild-type fish.
(4.10 MB TIF)

Figure S7 Keratinocytes and ionocytes of maternal-zygotic *epcam* mutants mutants express markers of terminal differentiation. To study whether EpCAM is required for terminal differentiation of epidermal cells, maternal/zygotic *epcam* mutants (MZ−/−; B,D) and wild-type controls (WT; A,C) were stained at 1 dpf for *ATPase1b1b* and *ATPase6v1al* transcripts, markers of two ionocytes subtypes, differentiated osmoregulatory skin cells that stem from the same pool of epidermal progenitors like keratinocytes (A,B), or at 3 dpf for keratin protein, a marker for differentiated basal keratinocytes of zebrafish larvae [74] (C,D). Mutants displayed normal signal intensities, normal numbers of ionocytes (A,B), and normal keratin distribution in basal cells (C,D), indicating that EpCAM is dispensable for the differentiation of epidermal cells. In addition, keratin distribution in primary neuromasts appeared normal (indicated by red arrows in C,D).
(4.51 MB TIF)

Text S1 Supporting Materials and Methods.
(0.03 MB DOC)

Video S1 Nomarki optics time-lapse movie of 2 days old wild-type embryo. Tail tip region ventral of notochord and directly posterior of the connecting flexure between caudal artery and caudal vein; anterior to the left; dorsal side up. Movies were taken at a Zeiss Axioplan microscope with a 40× water immersion lens; embryos were anesthetized, embedded in low melting agarose and imaged using Open Lab software (Improvision). Photos were taken every 30 sec and movie was generated at a rate of 10 frames/sec.
(4.03 MB MOV)

Video S2 Nomarki optics time-lapse movie of 2 days old MZ*epam* mutant embryo. The mutant has many more innate immune cells patrolling throughout the fin tissue. For details, see legend to Video S1.
(3.48 MB MOV)

## References

[1] Nakamura H, Yasuda M (1979). An electron microscopic study of periderm cell development in mouse limb buds.. Anat Embryol (Berl).

[2] M'Boneko V, Merker HJ (1988). Development and morphology of the periderm of mouse embryos (days 9–12 of gestation).. Acta Anat (Basel).

[3] Cui CY, Kunisada M, Esibizione D, Grivennikov SI, Piao Y (2007). Lymphotoxin-beta regulates periderm differentiation during embryonic skin development.. Hum Mol Genet.

[4] Vasilyev A, Liu Y, Mudumana S, Mangos S, Lam PY (2009). Collective cell migration drives morphogenesis of the kidney nephron.. PLoS Biol.

[5] Kimmel CB, Warga RM, Schilling TF (1990). Origin and organization of the zebrafish fate map.. Development.

[6] Le Guellec D, Morvan-Dubois G, Sire JY (2004). Skin development in bony fish with particular emphasis on collagen deposition in the dermis of the zebrafish (Danio rerio).. Int J Dev Biol.

[7] Warga RM, Kimmel CB (1990). Cell movements during epiboly and gastrulation in zebrafish.. Development.

[8] Kane D, Adams R (2002). Life at the edge: epiboly and involution in the zebrafish.. Results Probl Cell Differ.

[9] Solnica-Krezel L (2006). Gastrulation in zebrafish – all just about adhesion?. Curr Opin Genet Dev.

[10] Köppen M, Fernandez BG, Carvalho L, Jacinto A, Heisenberg CP (2006). Coordinated cell-shape changes control epithelial movement in zebrafish and Drosophila.. Development.

[11] Kane DA, McFarland KN, Warga RM (2005). Mutations in half baked/E-cadherin block cell behaviors that are necessary for teleost epiboly.. Development.

[12] Shimizu T, Yabe T, Muraoka O, Yonemura S, Aramaki S (2005). E-cadherin is required for gastrulation cell movements in zebrafish.. Mech Dev.

[13] Sonawane M, Carpio Y, Geisler R, Schwarz H, Maischein HM (2005). Zebrafish *penner*/*lethal giant larvae 2* functions in hemidesomosome formation, maintenance of cellular morphology and growth regulation in the developing basal epidermis.. Development.

[14] Kiener TK, Selptsova-Friedrich I, Hunziker W (2008). Tjp3/zo-3 is critical for epidermal barrier function in zebrafish embryos.. Dev Biol.

[15] Herlyn M, Steplewski Z, Herlyn D, Koprowski H (1979). Colorectal carcinoma-specific antigen: detection by means of monoclonal antibodies.. Proc Natl Acad Sci U S A.

[16] Koprowski H, Steplewski Z, Mitchell K, Herlyn M, Herlyn D (1979). Colorectal carcinoma antigens detected by hybridoma antibodies.. Somatic Cell Genet.

[17] Baeuerle PA, Gires O (2007). EpCAM (CD326) finding its role in cancer.. Br J Cancer.

[18] Trzpis M, McLaughlin PM, de Leij LM, Harmsen MC (2007). Epithelial cell adhesion molecule: more than a carcinoma marker and adhesion molecule.. Am J Pathol.

[19] Balzar M, Winter MJ, de Boer CJ, Litvinov SV (1999). The biology of the 17-1A antigen (Ep-CAM).. J Mol Med.

[20] Winter MJ, Nagelkerken B, Mertens AE, Rees-Bakker HA, Briaire-de Bruijn IH (2003). Expression of Ep-CAM shifts the state of cadherin-mediated adhesions from strong to weak.. Exp Cell Res.

[21] Schiechl H, Dohr G (1987). Immunohistochemical studies of the distribution of a basolateral-membrane protein in intestinal epithelial cells (GZ1-Ag) in rats using monoclonal antibodies.. Histochemistry.

[22] Klein CE, Cordon-Cardo C, Soehnchen R, Cote RJ, Oettgen HF (1987). Changes in cell surface glycoprotein expression during differentiation of human keratinocytes.. J Invest Dermatol.

[23] Cirulli V, Crisa L, Beattie GM, Mally MI, Lopez AD (1998). KSA antigen Ep-CAM mediates cell-cell adhesion of pancreatic epithelial cells: morphoregulatory roles in pancreatic islet development.. J Cell Biol.

[24] de Boer CJ, van Krieken JH, Janssen-van Rhijn CM, Litvinov SV (1999). Expression of Ep-CAM in normal, regenerating, metaplastic, and neoplastic liver.. J Pathol.

[25] Schmelzer E, Reid LM (2008). EpCAM expression in normal, non-pathological tissues.. Front Biosci.

[26] Trzpis M, McLaughlin PM, Popa ER, Terpstra P, van Kooten TG (2008). EpCAM homologues exhibit epithelial-specific but different expression patterns in the kidney.. Transgenic Res.

[27] Breuhahn K, Baeuerle PA, Peters M, Prang N, Tox U (2006). Expression of epithelial cellular adhesion molecule (Ep-CAM) in chronic (necro-)inflammatory liver diseases and hepatocellular carcinoma.. Hepatol Res.

[28] Trzpis M, McLaughlin PM, van Goor H, Brinker MG, van Dam GM (2008). Expression of EpCAM is up-regulated during regeneration of renal epithelia.. J Pathol.

[29] Gires O, Klein CA, Baeuerle PA (2009). On the abundance of EpCAM on cancer stem cells.. Nat Rev Cancer.

[30] Momburg F, Moldenhauer G, Hammerling GJ, Moller P (1987). Immunohistochemical study of the expression of a Mr 34,000 human epithelium-specific surface glycoprotein in normal and malignant tissues.. Cancer Res.

[31] Trzpis M, Bremer E, McLaughlin PM, de Leij LF, Harmsen MC (2008). EpCAM in morphogenesis.. Front Biosci.

[32] Jojovic M, Adam E, Zangemeister-Wittke U, Schumacher U (1998). Epithelial glycoprotein-2 expression is subject to regulatory processes in epithelial-mesenchymal transitions during metastases: an investigation of human cancers transplanted into severe combined immunodeficient mice.. Histochem J.

[33] Litvinov SV, Velders MP, Bakker HA, Fleuren GJ, Warnaar SO (1994). Ep-CAM: a human epithelial antigen is a homophilic cell-cell adhesion molecule.. J Cell Biol.

[34] Würfel J, Rosel M, Seiter S, Claas C, Herlevsen M (1999). Metastasis-association of the rat ortholog of the human epithelial glycoprotein antigen EGP314.. Oncogene.

[35] Litvinov SV, Balzar M, Winter MJ, Bakker HA, Briaire-de Bruijn IH (1997). Epithelial cell adhesion molecule (Ep-CAM) modulates cell-cell interactions mediated by classic cadherins.. J Cell Biol.

[36] Schmidt DS, Klingbeil P, Schnolzer M, Zoller M (2004). CD44 variant isoforms associate with tetraspanins and EpCAM.. Exp Cell Res.

[37] Ladwein M, Pape UF, Schmidt DS, Schnolzer M, Fiedler S (2005). The cell-cell adhesion molecule EpCAM interacts directly with the tight junction protein claudin-7.. Exp Cell Res.

[38] Kominsky SL, Argani P, Korz D, Evron E, Raman V (2003). Loss of the tight junction protein claudin-7 correlates with histological grade in both ductal carcinoma in situ and invasive ductal carcinoma of the breast.. Oncogene.

[39] Sauer T, Pedersen MK, Ebeltoft K, Naess O (2005). Reduced expression of Claudin-7 in fine needle aspirates from breast carcinomas correlate with grading and metastatic disease.. Cytopathology.

[40] Osta WA, Chen Y, Mikhitarian K, Mitas M, Salem M (2004). EpCAM is overexpressed in breast cancer and is a potential target for breast cancer gene therapy.. Cancer Res.

[41] Maetzel D, Denzel S, Mack B, Canis M, Went P (2009). Nuclear signalling by tumour-associated antigen EpCAM.. Nat Cell Biol.

[42] Munz M, Kieu C, Mack B, Schmitt B, Zeidler R (2004). The carcinoma-associated antigen EpCAM upregulates c-myc and induces cell proliferation.. Oncogene.

[43] Munz M, Zeidler R, Gires O (2005). The tumour-associated antigen EpCAM upregulates the fatty acid binding protein E-FABP.. Cancer Lett.

[44] Simon B, Podolsky DK, Moldenhauer G, Isselbacher KJ, Gattoni-Celli S (1990). Epithelial glycoprotein is a member of a family of epithelial cell surface antigens homologous to nidogen, a matrix adhesion protein.. Proc Natl Acad Sci U S A.

[45] Amsterdam A, Nissen RM, Sun Z, Swindell EC, Farrington S (2004). Identification of 315 genes essential for early zebrafish development.. Proc Natl Acad Sci U S A.

[46] Mills AA, Zheng B, Wang X-J, Vogel H, Roop DR, Bradley A (1999). *p63* is a *p53* homologue required for limb and epidermal morphogenesis.. Nature.

[47] Yang A, Schweitzer R, Sun D, Kaghad M, Walker N (1999). p63 is essential for regenerative proliferation in limb, craniofacial and epithelial development.. Nature.

[48] Bakkers J, Hild M, Kramer C, Furutani-Seiki M, Hammerschmidt M (2002). Zebrafish DeltaNp63 is a direct target of Bmp signaling and encodes a transcriptional repressor blocking neural specification in the ventral ectoderm.. Dev Cell.

[49] Lee H, Kimelman D (2002). A dominant-negative form of p63 is required for epidermal proliferation in zebrafish.. Dev Cell.

[50] Yang A, Kaghad M, Caput D, McKeon F (2002). On the shoulders of giants: p63, p73 and the rise of p53.. Trends Genet.

[51] Suh EK, Yang A, Kettenbach A, Bamberger C, Michaelis AH (2006). p63 protects the female germ line during meiotic arrest.. Nature.

[52] Gompel N, Cubedo N, Thisse C, Thisse B, Dambly-Chaudiere C (2001). Pattern formation in the lateral line of zebrafish.. Mech Dev.

[53] Villablanca EJ, Renucci A, Sapede D, Lec V, Soubiran F (2006). Control of cell migration in the zebrafish lateral line: implication of the gene “tumour-associated calcium signal transducer,” tacstd.. Dev Dyn.

[54] Thisse B, Pflumio S, Fürthauer M, Loppin B, Heyer V, Degrave A, Woehl R, Lux A, Steffan T, Charbonnier XQ, Thisse C (2001). Expression of the zebrafish genome during embryogenesis (NIH R01 RR15402)..

[55] Gong Z, Ju B, Wang X, He J, Wan H (2002). Green fluorescent protein expression in germ-line transmitted transgenic zebrafish under a stratified epithelial promoter from keratin8.. Dev Dyn.

[56] Carney TJ, von der Hardt S, Sonntag C, Amsterdam A, Topczewski J (2007). Inactivation of serine protease Matriptase1a by its inhibitor Hai1 is required for epithelial integrity of the zebrafish epidermis.. Development.

[57] Mathias JR, Dodd ME, Walters KB, Rhodes J, Kanki JP (2007). Live imaging of chronic inflammation caused by mutation of zebrafish Hai1.. J Cell Sci.

[58] Rhodes J, Hagen A, Hsu K, Deng M, Liu TX (2005). Interplay of pu.1 and gata1 determines myelo-erythroid progenitor cell fate in zebrafish.. Dev Cell.

[59] Herbomel P, Thisse B, Thisse C (1999). Ontogeny and behaviour of early macrophages in the zebrafish embryo.. Development.

[60] Meijer AH, van der Sar AM, Cunha C, Lamers GE, Laplante MA (2008). Identification and real-time imaging of a myc-expressing neutrophil population involved in inflammation and mycobacterial granuloma formation in zebrafish.. Dev Comp Immunol.

[61] Kiener TK, Sleptsova-Friedrich I, Hunziker W (2007). Identification, tissue distribution and developmental expression of tjp1/zo-1, tjp2/zo-2 and tjp3/zo-3 in the zebrafish, Danio rerio.. Gene Expr Patterns.

[62] Balzar M, Prins FA, Bakker HA, Fleuren GJ, Warnaar SO (1999). The structural analysis of adhesions mediated by Ep-CAM.. Exp Cell Res.

[63] Riethmüller G, Holz E, Schlimok G, Schmiegel W, Raab R (1998). Monoclonal antibody therapy for resected Dukes' C colorectal cancer: seven-year outcome of a multicenter randomized trial.. J Clin Oncol.

[64] Winter MJ, Nagtegaal ID, van Krieken JH, Litvinov SV (2003). The epithelial cell adhesion molecule (Ep-CAM) as a morphoregulatory molecule is a tool in surgical pathology.. Am J Pathol.

[65] Hardison AL, Lichten L, Banerjee-Basu S, Becker TS, Burgess SM (2005). The zebrafish gene claudinj is essential for normal ear function and important for the formation of the otoliths.. Mech Dev.

[66] Lachnit M, Kur E, Driever W (2008). Alterations of the cytoskeleton in all three embryonic lineages contribute to the epiboly defect of Pou5f1/Oct4 deficient MZspg zebrafish embryos.. Dev Biol.

[67] Hammerschmidt M, Wedlich D (2008). Regulated adhesion as a driving force of gastrulation movements.. Development.

[68] Yap AS, Crampton MS, Hardin J (2007). Making and breaking contacts: the cellular biology of cadherin regulation.. Curr Opin Cell Biol.

[69] Ulrich F, Krieg M, Schotz EM, Link V, Castanon I (2005). Wnt11 functions in gastrulation by controlling cell cohesion through Rab5c and E-cadherin.. Dev Cell.

[70] Ogata S, Morokuma J, Hayata T, Kolle G, Niehrs C (2007). TGF-beta signaling-mediated morphogenesis: modulation of cell adhesion via cadherin endocytosis.. Genes Dev.

[71] Winter MJ, Cirulli V, Briaire-de Bruijn IH, Litvinov SV (2007). Cadherins are regulated by Ep-CAM via phosphaditylinositol-3 kinase.. Mol Cell Biochem.

[72] Kubota K, Furuse M, Sasaki H, Sonoda N, Fujita K (1999). Ca(2+)-independent cell-adhesion activity of claudins, a family of integral membrane proteins localized at tight junctions.. Curr Biol.

[73] Turksen K, Troy TC (2004). Barriers built on claudins.. J Cell Sci.

[74] Webb AE, Driever W, Kimelman D (2008). psoriasis regulates epidermal development in zebrafish.. Dev Dyn.

[75] Linnenbach AJ, Seng BA, Wu S, Robbins S, Scollon M (1993). Retroposition in a family of carcinoma-associated antigen genes.. Mol Cell Biol.

[76] Amsterdam A, Burgess S, Golling G, Chen W, Sun Z (1999). A large-scale insertional mutagenesis screen in zebrafish.. Genes Dev.

[77] Cooper MS, Szeto DP, Sommers-Herivel G, Topczewski J, Solnica-Krezel L (2005). Visualizing morphogenesis in transgenic zebrafish using BODIYP TR methyl ester dye as a vital counterstain for GFP.. Dev Dyn.

[78] Huang H, Marsh-Armstrong N, Brown DD (1999). Metamorphosis is inhibited in transgenic Xenopus laevis tadpoles that overexpress type III deiodinase.. Proc Natl Acad Sci U S A.

[79] von der Hardt S, Bakkers J, Inbal A, Carvalho L, Solnica-Krezel L (2007). The Bmp gradient of the zebrafish gastrula guides migrating lateral cells by regulating cell-cell adhesion.. Curr Biol.

[80] Pouthas F, Girard P, Lecaudey V, Ly TB, Gilmour D (2008). In migrating cells, the Golgi complex and the position of the centrosome depend on geometrical constraints of the substratum.. J Cell Sci.

[81] Yamada S, Pokutta S, Drees F, Weis WI, Nelson WJ (2005). Deconstructing the cadherin-catenin-actin complex.. Cell.

[82] Huber O, Kemler R, Langosch D (1999). Mutations affecting transmembrane segment interactions impair adhesiveness of E-cadherin.. J Cell Sci.

[83] Hammerschmidt M, Pelegri F, Mullins MC, Kane DA, van Eeden FJ (1996). dino and mercedes, two genes regulating dorsal development in the zebrafish embryo.. Development.

[84] Ruf P, Gires O, Jager M, Fellinger K, Atz J (2007). Characterisation of the new EpCAM-specific antibody HO-3: implications for trifunctional antibody immunotherapy of cancer.. Br J Cancer.

[85] Bjork P, Jonsson U, Svedberg H, Larsson K, Lind P (1993). Isolation, partial characterization, and molecular cloning of a human colon adenocarcinoma cell-surface glycoprotein recognized by the C215 mouse monoclonal antibody.. J Biol Chem.

[86] Nasevicius A, Ekker SC (2000). Effective targeted gene ‘knockdown’ in zebrafish.. Nat Genet.

[87] Montero JA, Carvalho L, Wilsch-Brauninger M, Kilian B, Mustafa C (2005). Shield formation at the onset of zebrafish gastrulation.. Development.

[88] Kane DA, Kimmel CB (1993). The zebrafish midblastula transition.. Development.

