# Supplementary figures and images for: The Epithelial Cell Adhesion Molecule EpCAM Is Required for Epithelial Morphogenesis and Integrity during Zebrafish Epiboly and Skin Development
Source: PLoS Genet. 2009 Jul 17;5(7):e1000563. doi: 10.1371/journal.pgen.1000563 (PMC2700972; doi:10.1371/journal.pgen.1000563)

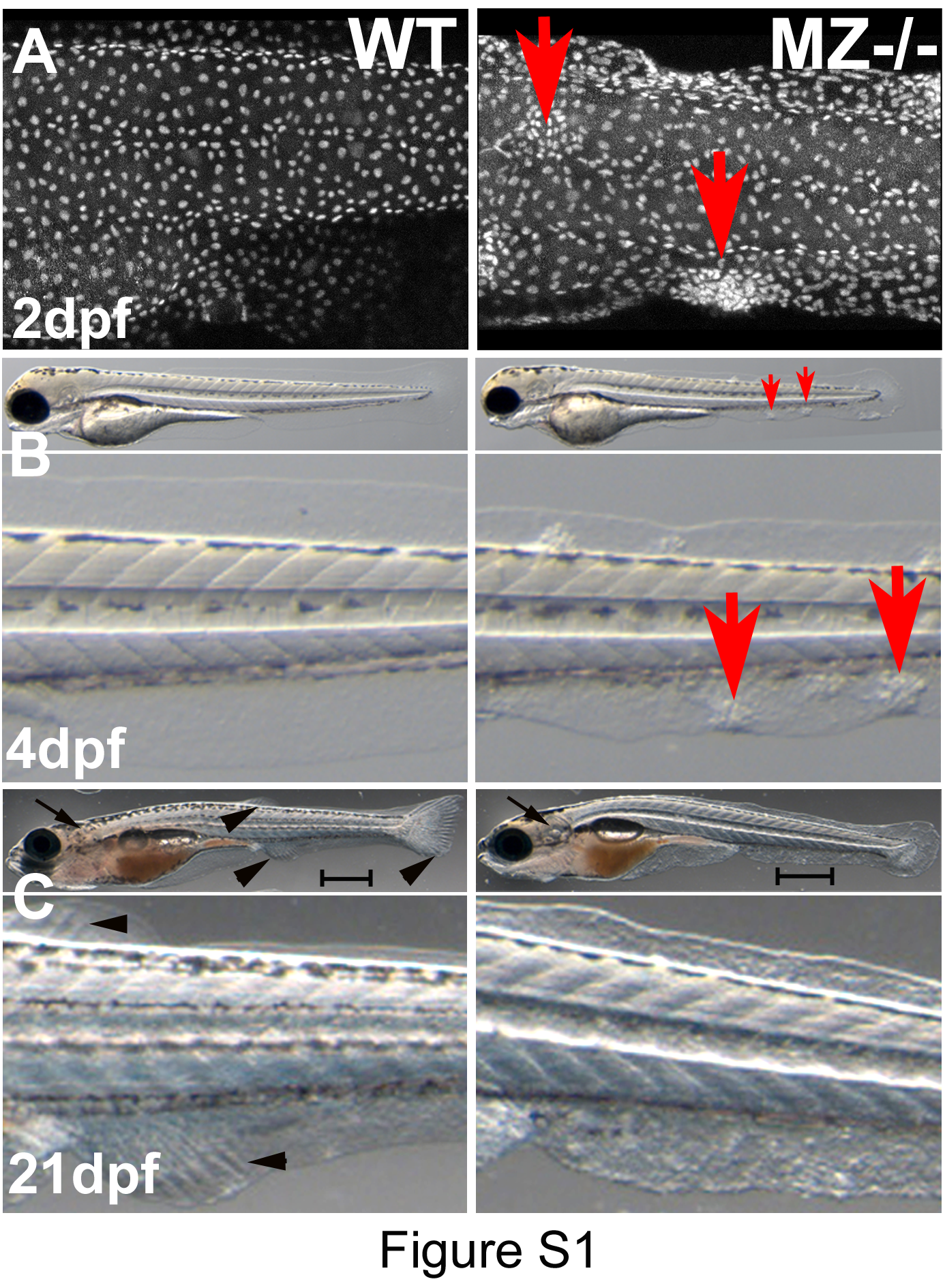

Supplement: Figure S1 — epcam mutants form skin cell aggregates. (A) Confocal image (merged Z-stack) of basal keratinocytes of wild type control (WT) and maternal/zygotic epcam mutant (MZ−/−) embryos at 2 days post fertilization (dpf), after anti-p63 immunostaining of basal keratinocytes. Basal cell aggregates of mutant are indicated by red arrows. (B,C) Overviews (upper panels) and magnified views of tail region (lower panels) of live WT (left panels) and MZ−/− mutants (right panels) at 4 days post fertilization (dpf) (B) and 21 dpf, when the skin becomes multi-layered (C). The epidermal aggregates of mutants persist during further larval development (B; indicated by red arrows), whereas they are less prominent at 21 dpf (C). In addition, otoliths have recovered and acquired normal size at 21 dpf (C; indicated by black arrows). However, the skin of the mutant has a rougher morphology, the mutant is of reduced size (C; see different lengths of scale bars = 1 mm), and fin development is delayed, as judged by the less advanced fin ray formation in the developing unpaired anal, dorsal and tail fins (C; indicated by black arrowheads in wild-type animal). Further analyses have to reveal whether this is a skin/fin-specific defect, or a consequence of generally delayed development, growth and metamorphosis. (3.16 MB TIF) [file pgen.1000563.s001.tif]

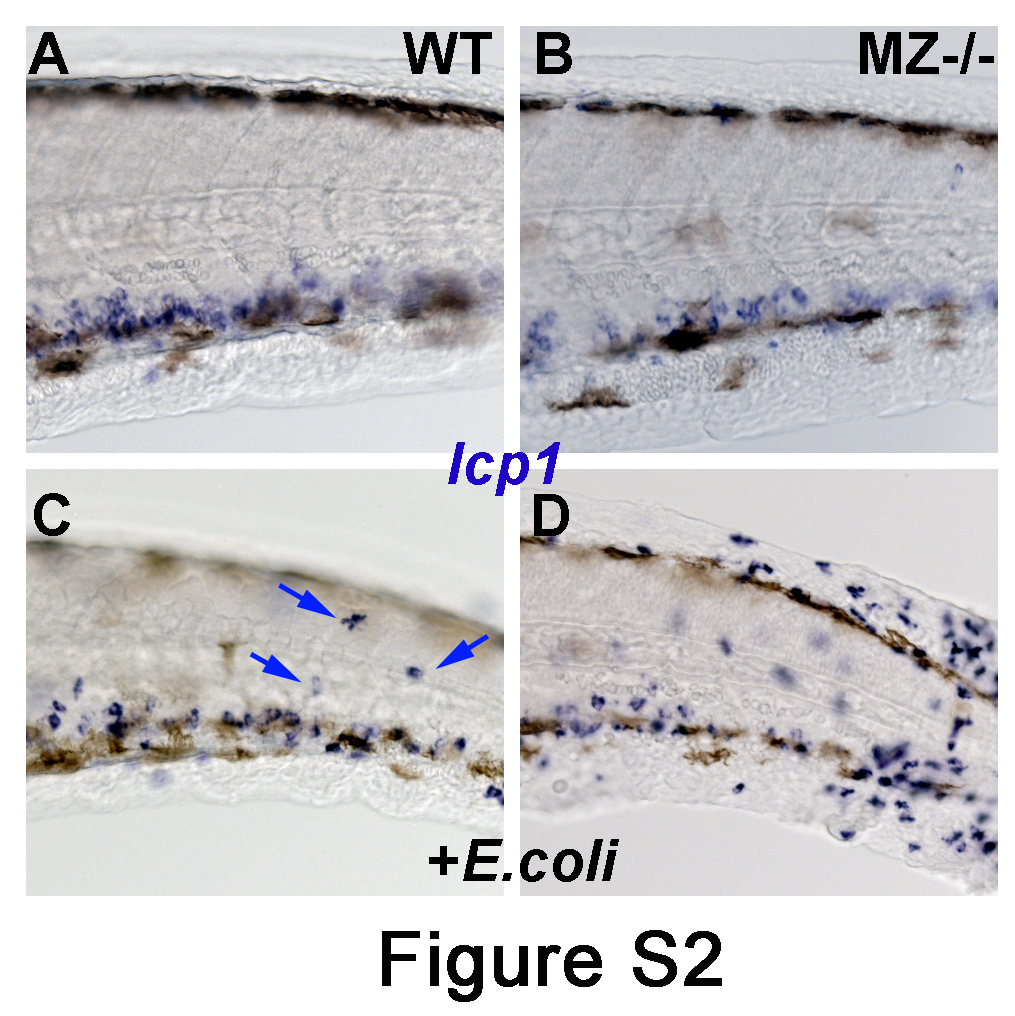

Supplement: Figure S2 — MZepcam mutants display increased susceptibility to cutaneous infections. All panels show lateral views on the tail region of embryos at 2 dpf, after whole mount in-situ hybridization for leukocyte-specific-plastin (lcp1) mRNA, a marker for leukocytes (macrophages and neutrophiles) [59]. (A,B) In wild-type (WT; A) and maternal-zygotic epcam mutants (MZ−/−; B) kept in semi sterile medium, leukocytes are found mainly in the blood vessels. (C,D) Addition of E. coli to the incubation medium stimulates the presence of innate immune cells in the skin (indicated by blue arrows in wild-type fish; C). Skin inflammation is much stronger in the challenged mutant (D) than in the wild-type control (C), suggesting that the mutant is more susceptible to cutaneous infections. (1.33 MB TIF) [file pgen.1000563.s002.tif]

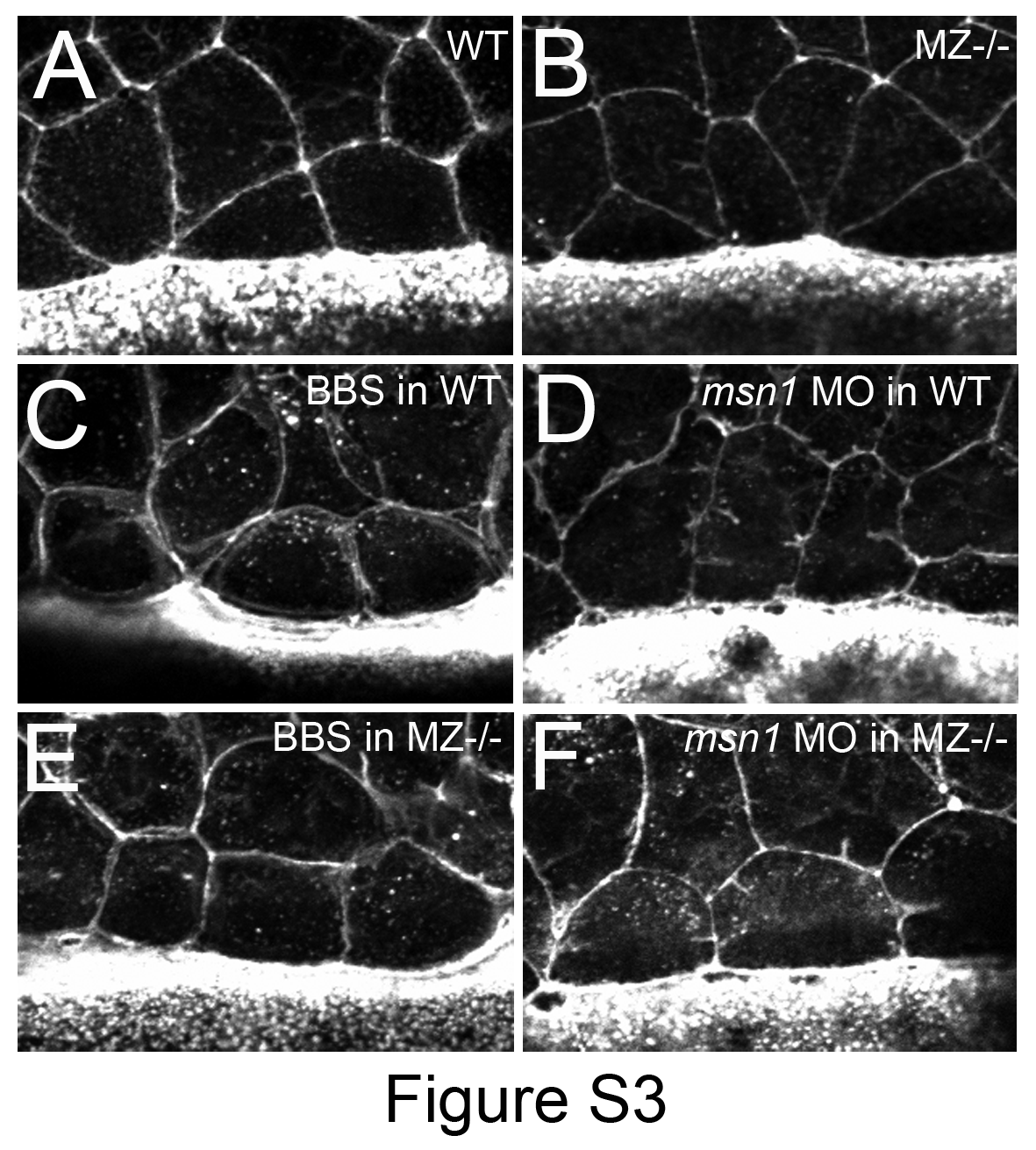

Supplement: Figure S3 — In contrast to loss of msn1 or treatment with blebbistatin, loss of epcam does not compromise the constriction of marginal EVL cells. (A–F) Fluorescent confocal images of phalloidin stained maternal zygotic-EpCAM mutant (MZ−/−) and wild type (WT) embryos at 70% epiboly. The leading EVL cells of both, mutant and WT embryos are constricted by an actin-myosin string as indicated by the presence of cells with shorter leading than trailing sides (A,B). This is in contrary to the release of constriction caused by in wild-type embryos upon treatment with 15 ug/ml blebbistatin (C) or injection of msn1 MO (D) [10], suggesting that the EVL epiboly defects of MZepcam and msn1 mutants have a different cellular basis, and that the phenotype of MZepcam mutants is not caused by reduced actin-myosin constriction at the EVL-YSL interface. Consistent with this notion, injection of msn1 MO or blebbistatin treatment in MZepcam mutants, while releasing marginal constrictions (E,F), failed to synergistically enhance the EVL epiboly defects, but rather had pure additive effects (data not shown). Another possibility would have been that the epiboly defects of MZepcam mutants are due to increased/precocious, rather than reduced actin-myosin constriction at the EVL-YSL interface. However, applying increasingly lower amounts of msn1 MO or blebbistatin to MZepcam mutants, we never obtained an alleviation of the EVL epiboly defects (judged by the extrusion of the vegetal-most part of the yolk at late gastrula stages; compare with Figure 5B), also making this possibility very unlikely. (1.59 MB TIF) [file pgen.1000563.s003.tif]

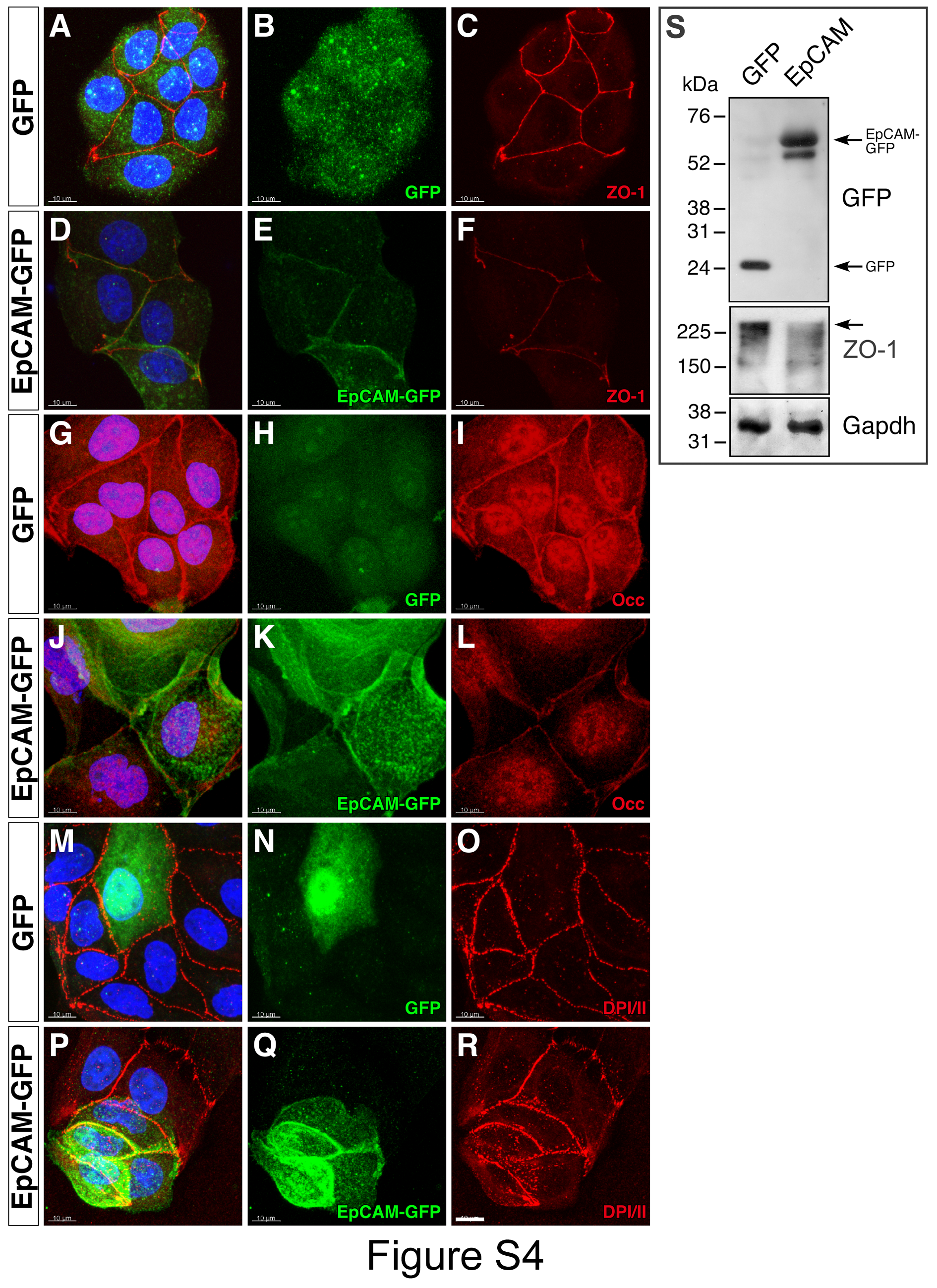

Supplement: Figure S4 — Overexpression of EpCAM in MDCK cells causes reduced levels of tight junction proteins. MDCK cells were transfected with expression vectors driving either GFP (A–C, G–I, M–O) or zebrafish EpCAM-GFP expression (D–F, J–L, P–R), plated on cover slips after FACS sorting one day after transfection, and stained for cell-cell junctional markers 48 hours after plating. Processed confocal images are shown as merges of entire Z-stacks. Transfectants were detected by GFP (B, H, N) or EpCAM-GFP expression (E, K, Q). Protein localization was analyzed with antibodies against Tjp1/ZO-1 (C, F), Occludin (Occ) (I, L), or Desmoplakin 1 and 2 (DPI/II) (O, R); panels (A,D,G,J,M,P) show overlays with GFP fluorescence. DAPI-stained nuclei are shown in blue (A,D,G,J,M,P); scale bar, 10 µm. Fluorescent labeling of key components of tight junctions reveals a reduction in membrane localization of Tjp1 in EpCAM- (F) versus GFP-transfected (C) cells. Similarly, Occludin staining is reduced in EpCAM expressing MDCK cells (I, L). However, proteins localizing to adherens junctions or desmosomes are not altered in expression or cellular distribution, as shown for DPI/II (O, R). This indicates that EpCAM expression in MDCK cells leads to a modification in apical junction complex assembly, resulting in a reduction of key tight junction proteins at the plasma membrane. (S) Immunoblot of lysats from FACS-sorted MDCK cells transfected with EpCAM-GFP (right lane) or, as control, GFP (left lane). EpCAM-GFP and GFP are expressed at comparable levels (upper panel). Expression of EpCAM-GFP leads to a significant in Tjp1/ZO1 protein levels (middle panel; arrow indicate full-length Tjp1/ZO1 protein). Gapdh was used as loading control (lower panel). (14.76 MB TIF) [file pgen.1000563.s004.tif]

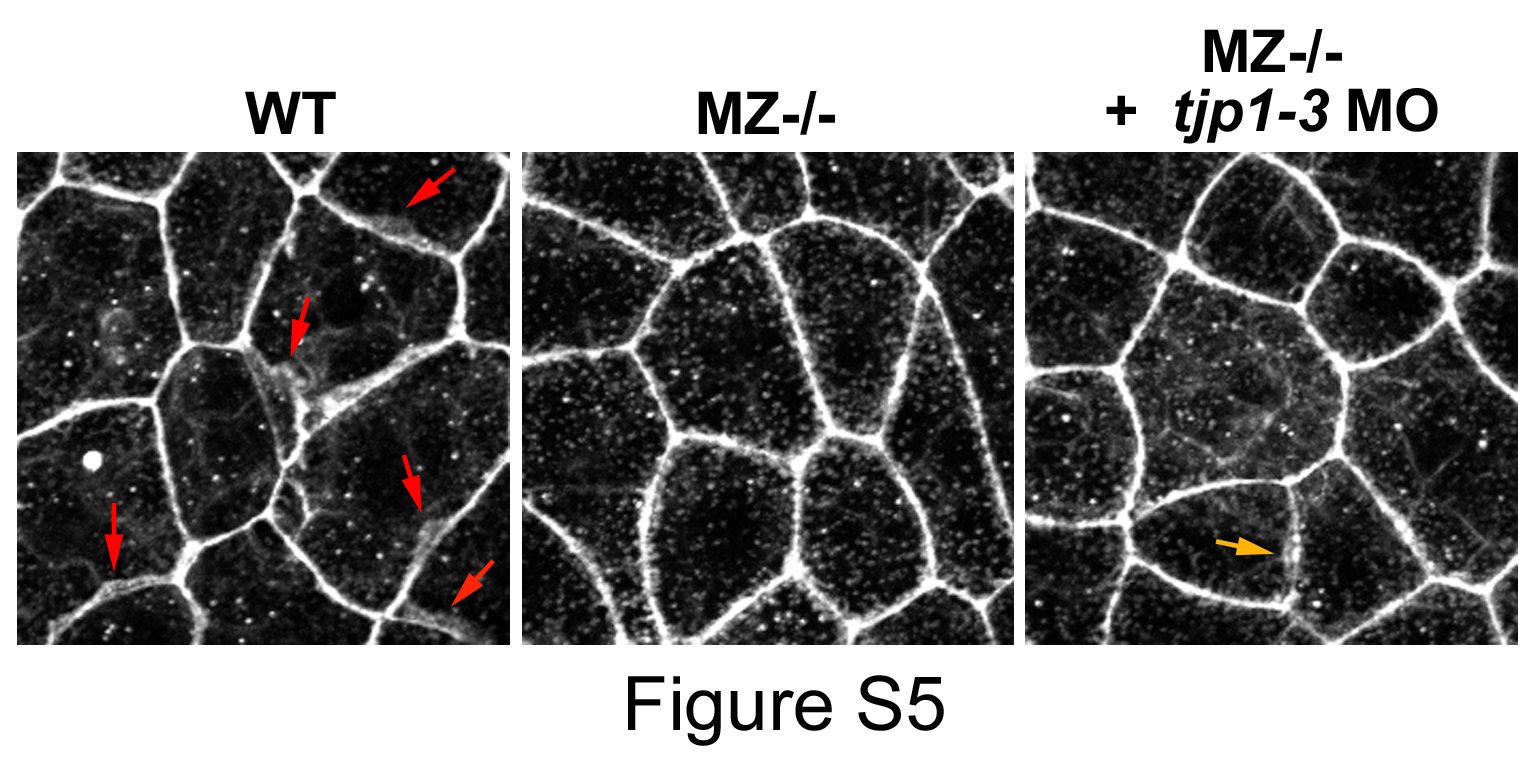

Supplement: Figure S5 — Knockdown of tight junction proteins 1–3 fails to restore ruffle formation in epcam mutants. Fluorescent confocal images of phalloidin stained wild type (WT) and maternal-zygotic EpCAM mutant (MZ−/−) embryos. Single and collective knock down of tight junction proteins 1–3 via antisense morpholino oligonucleotides (MO) injections into MZ−/− embryo (panel 2) failed to restore the protrusive activity of the mutant EVL cells back to the WT levels (panel 1, red arrows point to ruffles). If at all, rescue was very minor (orange arrow in panel 3 points to one of the few and rather small ruffles found in 12 investigated tjp1–3 MO-injected embryos). (3.63 MB TIF) [file pgen.1000563.s005.tif]

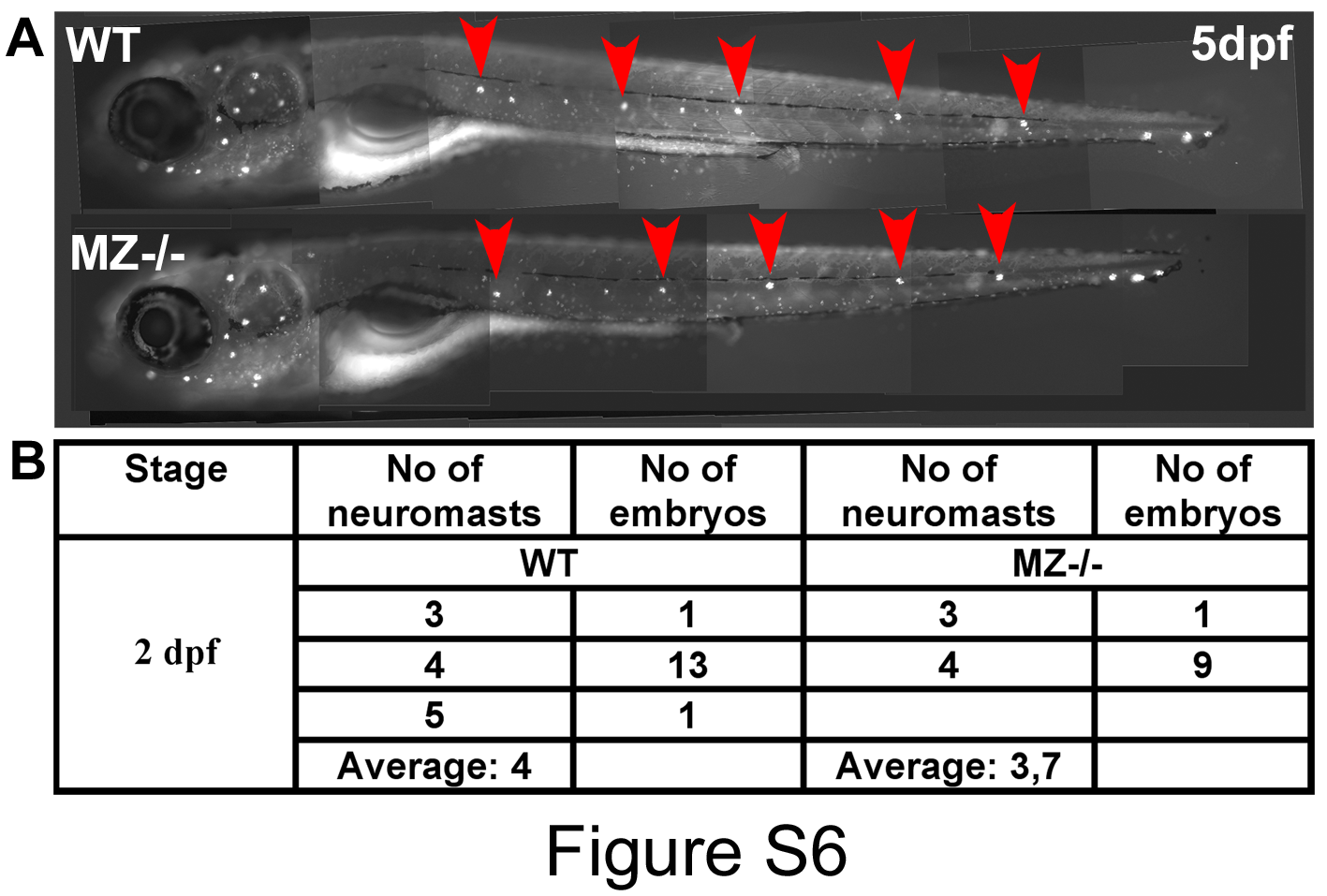

Supplement: Figure S6 — Maternal-zygotic epcam mutants have unaltered numbers of deposited primary neuromasts. (A) 5 dpf old maternal-zygotic epcam mutants (MZ−/− ) and wild-type (WT) controls at 120 hpf, stained with 4-Di-2-ASP for vital labeling of differentiated hair cells in the larval lateral line. The red arrowheads point the deposited primary neuromasts. (B) Numbers of deposited primary neuromasts in WT control and MZ−/− mutants embryos at 2 dpf. Neuromasts were stained by their endogenous alkaline phosphatase activity [80]. Contrary to published data obtained from epcam morpholino studies [53], we could not detect significant differences in the numbers of deposited neuromasts between mutant and wild-type fish. (4.10 MB TIF) [file pgen.1000563.s006.tif]

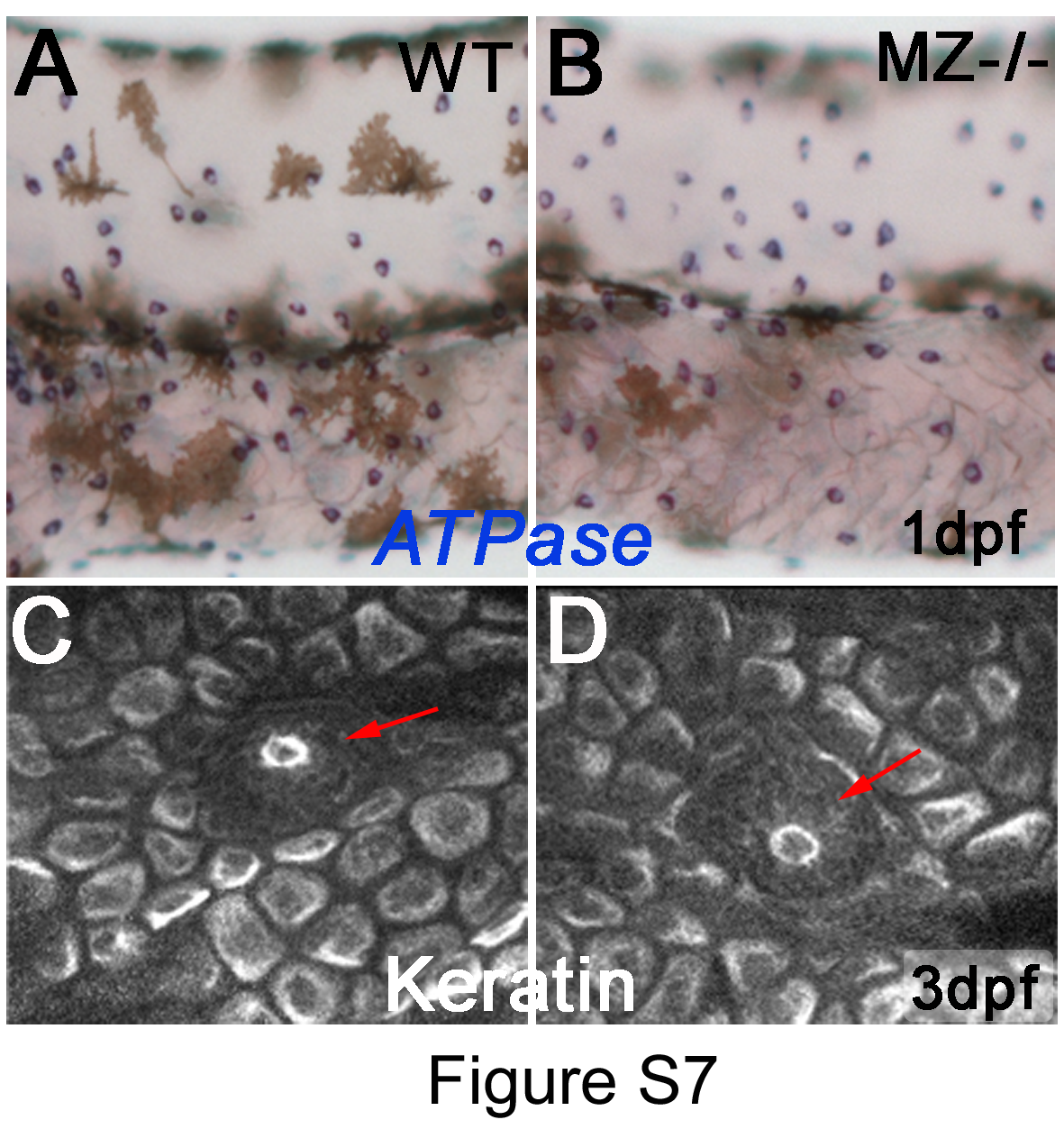

Supplement: Figure S7 — Keratinocytes and ionocytes of maternal-zygotic epcam mutants mutants express markers of terminal differentiation. To study whether EpCAM is required for terminal differentiation of epidermal cells, maternal/zygotic epcam mutants (MZ−/−; B,D) and wild-type controls (WT; A,C) were stained at 1 dpf for ATPase1b1b and ATPase6v1al transcripts, markers of two ionocytes subtypes, differentiated osmoregulatory skin cells that stem from the same pool of epidermal progenitors like keratinocytes (A,B), or at 3 dpf for keratin protein, a marker for differentiated basal keratinocytes of zebrafish larvae [74] (C,D). Mutants displayed normal signal intensities, normal numbers of ionocytes (A,B), and normal keratin distribution in basal cells (C,D), indicating that EpCAM is dispensable for the differentiation of epidermal cells. In addition, keratin distribution in primary neuromasts appeared normal (indicated by red arrows in C,D). (4.51 MB TIF) [file pgen.1000563.s007.tif]
