# Supplementary material for: The Epithelial Cell Adhesion Molecule EpCAM Is Required for Epithelial Morphogenesis and Integrity during Zebrafish Epiboly and Skin Development
Source: PLoS Genet. 2009 Jul 17;5(7):e1000563. doi: 10.1371/journal.pgen.1000563 (PMC2700972; doi:10.1371/journal.pgen.1000563)
Supplement: Text S1 — Supporting Materials and Methods. (0.03 MB DOC) [file pgen.1000563.s008.doc]

**Supporting Information**

**Materials and Methods**

**Vital staining of hair cells**

For vital labeling of hair cells, larvae were immersed in a 200 μM solution of 4-(4-(diethylamino)styryl)-N-methylpyridinium iodide (4-Di-2-ASP; Molecular Probes) for 1 minute at room temperature in the dark. Treated larvae were washed briefly to remove excess fluorophore, anesthetized with 3-aminobenzoic acid ethyl ester, and mounted in 3% methylcellulose on a glass slide.

**MDCK cell experiments: transfection, immunofluorescence labeling and immunoblotting**

The following primary antibodies were used in 1:100 to 1:200 dilutions. Mouse anti-GFP (Roche, 11814460001), rabbit anti-ZO-1 (Zymed, 61-7300), rabbit anti-Occludin (Zymed, 71-1500, rabbit anti-Desmoplakin 1/2 (Pasdar and Nelson 1988), rabbit anti-E-cadherin (gp84) (Vestweber and Kemler 1984), monoclonal mouse anti-GADPH (Chemicon). Secondary species-specific alexa-fluorochrome-conjugated or horse reddish peroxidase-conjugated antibodies were purchased from Molecular Probes or Roche and used at 1:200.

MDCK (madine-darby canine kidney) cells were cultured under standard conditions in DMEM with 10% FCS. Cells were plated in 6 cm dishes (2x10^5^) and transfected after 24 hours with 2 µg of pXLT-GFP or pXLT-EpCAM-GFP. 24 hours later GFP-positive cells were FACS-sorted and plated on collagenIV-coated cover slips. Cells on cover slips were fixed with Methanol after 48 h, washed in PBS and incubated with primary antibodies for one hour. After removing unbound primary antibody by several washing steps with PBS, cover slips were incubated with alexa488 or alexa594-fluorochrome-conjugated antibodies for 30 min, washed with PBS and counter-stained with DAPI (1:1000, Molecular Probes, D3571) to visualize nuclei. Confocal microscopy was carried out with a Leica TCS SP2 UV laser scan head attached to a Leica DM IRE2 inverted microscope equipped with Leica Confocal software. Image processing was performed using IMARIS imaging software version 6.1 (Bitplane AG).

For immunoblotting, MDCK cells were lysed one day after replating at 50% cofluency with 200µl 1xSDS-LB (50 mM Tris-HCL pH6.8; 2% SDS; 10% Glycerol; 0.1% Brome phenol blue; 5% beta-mercaptoethanol). After sonication for 15 sec, samples were boiled at 100°C for 5 min, and 1/10 of the lysate was separated per lane via 8-12% SDS-PAGE. Primary antibodies (see above) were used at 1:200 dilution.

**In situ hybridizations and antibody staining**.

Anti-sense in situ probes for ATPase1b1b and ATPase6v1al subunits, markers for different subtypes of larval skin ionocytes, were generated and used as described (Jänicke et al. 2007). Pan-keratin antibody was used as described (Sonawane et al. 2005).

**References for Supporting Information:**

Jänicke M, Carney TJ, Hammerschmidt M (2007) Foxi3 transcription factors and Notch signaling control the formation of skin ionocytes from epidermal precursors of the zebrafish embryo. Dev Biol 307(2): 258-271.

Sonawane M, Carpio Y, Geisler R, Schwarz H, Maischein HM et al. (2005) Zebrafish *penner*/*lethal giant larvae* *2* functions in hemidesomosome formation, maintenance of cellular morphology and growth regulation in the developing basal epidermis. Development 132: 3255-3265.

Vestweber D, Kemler R (1984) Rabbit antiserum against a purified surface glycoprotein decompacts mouse preimplantation embryos and reacts with specific adult tissues. Exp Cell Res 152(1): 169-178.
